# Association of human height-related genetic variants with familial short stature in Han Chinese in Taiwan

**DOI:** 10.1038/s41598-017-06766-z

**Published:** 2017-07-25

**Authors:** Ying-Ju Lin, Wen-Ling Liao, Chung-Hsing Wang, Li-Ping Tsai, Chih-Hsin Tang, Chien-Hsiun Chen, Jer-Yuarn Wu, Wen-Miin Liang, Ai-Ru Hsieh, Chi-Fung Cheng, Jin-Hua Chen, Wen-Kuei Chien, Ting-Hsu Lin, Chia-Ming Wu, Chiu-Chu Liao, Shao-Mei Huang, Fuu-Jen Tsai

**Affiliations:** 1 Genetic Center, Department of Medical Research, China Medical University Hospital, Taichung, Taiwan; 2 School of Chinese Medicine, China Medical University, Taichung, Taiwan; 3 Graduate Institute of Integrated Medicine, China Medical University, Taichung, Taiwan; 4 Center for Personalized Medicine, China Medical University Hospital, Taichung, Taiwan; 5 Children’s Hospital of China Medical University, Taichung, Taiwan; 6 Department of Pediatrics, Buddhist Tzu Chi General Hospital, Taipei Branch, Taipei Taiwan; 7 Graduate Institute of Biomedical Sciences, China Medical University, Taichung, Taiwan; 8 Institute of Biomedical Sciences, Academia Sinica, Taipei, Taiwan; 9 Graduate Institute of Biostatistics, School of Public Health, China Medical University, Taichung, Taiwan; 10 Biostatistics Center and School of Public Health, Taipei Medical University, Taipei, Taiwan; 11 National Applied Research Laboratories, National Center for High-performance Computing, Hsinchu, Taiwan; 12 Department of Biotechnology and Bioinformatics, Asia University, Taichung, Taiwan

## Abstract

Human height can be described as a classical and inherited trait model. Genome-wide association studies (GWAS) have revealed susceptible loci and provided insights into the polygenic nature of human height. Familial short stature (FSS) represents a suitable trait for investigating short stature genetics because disease associations with short stature have been ruled out in this case. In addition, FSS is caused only by genetically inherited factors. In this study, we explored the correlations of FSS risk with the genetic loci associated with human height in previous GWAS, alone and cumulatively. We systematically evaluated 34 known human height single nucleotide polymorphisms (SNPs) in relation to FSS in the additive model (*p* < 0.00005). A cumulative effect was observed: the odds ratios gradually increased with increasing genetic risk score quartiles (*p* < 0.001; Cochran-Armitage trend test). Six affected genes—*ZBTB38*, *ZNF638*, *LCORL*, *CABLES1*, *CDK10*, and *TSEN15*—are located in the nucleus and have been implicated in embryonic, organismal, and tissue development. In conclusion, our study suggests that 13 human height GWAS-identified SNPs are associated with FSS risk both alone and cumulatively.

## Introduction

Human height can be described as a classical, inherited, and polygenic trait model. The ultimate phenotype represents the outcome of genetics and developmental biology, including the enlargement of bone length and the sizes of tissues and organ sizes. Undoubtedly, the developmental processes may be affected by environmental factors, including nutrition^1^, exercise^2^, diseases^3–5^, and infections^6, 7^. However, ~80% of the variance in height among individuals can also be explained by genetic variation^8–10^. Modern high-density genotyping platforms have now made it possible to identify candidate genes across the whole genome in relation to complex traits and diseases^11–13^.

Genetic factors that contribute to the genetic architecture of human height are widely identified by means of genome-wide screening methods^14–24^. Human height-related susceptibility loci related to the protein tyrosine phosphatase family, insulin-like growth factor, and skeletal growth have been reported in Asian populations^18, 19, 21, 22, 25^. In addition, predisposing loci determining height have also been discovered in European populations in genes related to skeletal development, mitosis, fibroblast growth factors, the WNT/β-catenin pathway, chondroitin sulfate-related genes, mammalian target of rampamycin, osteoglycin, binding of hyaluronic acid, Hedgehog signaling, the extracellular matrix, and cancer-associated pathways^14–17^. The results of genome-wide association studies (GWAS) have thus contributed new information on the susceptibility loci related to human height and provide insights into the extreme polygenic nature of human height.

Short stature is defined as a body height below the 3^rd^ percentile for the corresponding age and gender of the population. Among people affected by short stature, 37% have familial short stature (FSS), 27% have a constitutional growth delay, 17% have both FSS and a constitutional growth delay, 9% have a systemic disease, 5% have an endocrine condition, and the cause is unknown in the remaining 5% of cases^26–28^. Representing the majority of cases of short stature, FSS is characterized by a height below the 3^rd^ percentile in the population, a normal annual growth rate, normal bone age, family history of short stature, normal onset of puberty, and normal results of biochemical analyses. FSS is also called “genetic short stature” because the growth conditions are normal but the body height is below the 3^rd^ percentile and there is a family history of short stature. FSS represents a very suitable study object for research on short stature genetics because disease association has been ruled out for the short stature in FSS. FSS is caused only by genetically inherited factors. Thus, we initiated a pilot study to search for genetic loci that may predispose individuals to FSS. We explored the associations of FSS risk in Taiwan with genetic loci that have been related to human height according to previous GWAS, both alone and cumulatively.

## Results

### GWAS-determined genetic variants related to human height and FSS risk in the Han Chinese population of Taiwan

To identify the susceptible single nucleotide polymorphisms (SNPs) associated with FSS risk, we chose genetic SNPs that have been related to human height according to published GWAS, which were considered to be the closest genes potentially associated with FSS. There were 1,033 genetic SNPs reported in 14 human height GWAS conducted to date (Table S1). Of these, 34 SNPs were ultimately identified as candidate susceptibility SNPs for FSS according to the selection criteria [call rates of genotyping for both FSS and controls >95%, genotypes for genetic variants in controls conforming to Hardy-Weinberg equilibrium (*p* > 0.05), and *p*-value for the additive model <5.00E-05 (0.05/1,033)]. The 34 SNPs in 15 closest genes identified in human height GWAS are summarized in Table 1 and Supplementary Table S2. As shown in Table 1, the minor allele frequencies (MAFs) of these 34 genetic SNPs were similar to the MAFs in Han Chinese from Beijing (CHB) NCBI GRCh38.p2 assembly) from the online database of the dbSNP website (HAPMAP-CHB; http://www.ncbi.nlm.nih.gov/projects/SNP/index.html). The pair-wise linkage disequilibrium (LD) between the 34 SNPs was also estimated (Supplementary Tables S2–S5 and Figure S1).

**Table 1:** Information of genetic SNPs identified from GWAS of human height.

No.	rs ID	Gene	Chr.	Position	MAF (Allele)^a^	MAF (%) in CHB^a^	MAF (%) in controls	Call rate (%)	*P* for controls HWE
1	rs1926872	*COLGALT2*	1	184049341	G	47.1%	51.5%	100.0%	0.34
2	rs1046934	*TSEN15*	1	184054395	C	47.1%	51.8%	100.0%	0.35
3	rs3791679	*EFEMP1*	2	55869757	T	20.7%	24.9%	99.8%	0.08
4	rs3791675	*EFEMP1*	2	55884174	G	22.2%	25.3%	100.0%	0.26
5	rs3771381	*ZNF638*	2	71333535	T	42.7%	39.7%	100.0%	0.52
6	rs10935120	*CEP63*	3	134514250	A	18.9%	15.0%	99.8%	0.85
7	rs6440003	*ZBTB38*	3	141375367	A	35.6%	34.8%	100.0%	0.21
8	rs7632381	*ZBTB38*	3	141387221	C	34.4%	35.6%	100.0%	0.24
9	rs1344672	*ZBTB38*	3	141406863	C	33.3%	35.5%	100.0%	0.29
10	rs9825379	*ZBTB38*	3	141418193	A	17.1%	19.9%	99.9%	0.40
11	rs7678436	*DCAF16*	4	17796343	A	34.1%	32.4%	99.6%	0.69
12	rs16895802	*NCAPG*	4	17814266	G	12.2%	12.0%	100.0%	0.60
13	rs6842303	*LCORL*	4	17852432	T	45.0%	47.4%	100.0%	0.86
14	rs13131350	*LCORL*	4	17875864	G	22.2%	24.6%	100.0%	0.69
15	rs16896276	*LCORL*	4	18013533	A	46.7%	47.4%	100.0%	0.51
16	rs2011603	*LCORL*	4	18023861	G	45.1%	47.9%	100.0%	0.57
17	rs17720281	*NA*	4	144622624	T	20.0%	24.9%	100.0%	0.64
18	rs6845999	*HHIP*	4	144644674	T	26.8%	24.7%	99.9%	0.66
19	rs4240326	*ANAPC10*	4	144918112	A	36.7%	30.0%	100.0%	0.55
20	rs6823268	*ANAPC10*	4	145061411	G	17.1%	17.3%	100.0%	0.31
21	rs4733724	*GSDMC*	8	129711482	A	30.5%	28.2%	100.0%	0.10
22	rs6470764	*GSDMC*	8	129713419	C	33.0%	28.2%	100.0%	0.10
23	rs10858250	*QSOX2*	9	136227369	G	24.7%	23.4%	100.0%	0.90
24	rs12338076	*QSOX2*	9	136229894	C	32.4%	31.4%	99.9%	0.44
25	rs2401171	*ADAMTSL3*	15	83888924	T	29.1%	23.5%	100.0%	0.09
26	rs10906982	*ADAMTSL3*	15	83899406	T	28.9%	23.7%	99.8%	0.07
27	rs7183263	*ADAMTSL3*	15	83904289	T	29.1%	23.4%	100.0%	0.10
28	rs11259936	*ADAMTSL3*	15	83911830	A	29.1%	23.4%	100.0%	0.10
29	rs4842838	*ADAMTSL3*	15	83913372	G	28.9%	23.3%	99.8%	0.08
30	rs258324	*CDK10*	16	89687847	A	34.4%	32.5%	100.0%	0.50
31	rs4800452	*CABLES1*	18	23147647	C	17.6%	16.9%	100.0%	0.50
32	rs4369779	*CABLES1*	18	23155444	T	15.8%	14.4%	99.9%	0.53
33	rs4308051	*CABLES1*	18	23155497	T	15.8%	14.4%	100.0%	0.50
34	rs8094261	*CABLES1*	18	23166764	G	13.4%	14.8%	100.0%	0.44

Abbreviations: SNP, single nucleotide polymorphism; GWAS, genome-wide association studies; chr., chromosome; MAF, minor allele frequency; CHB, Han Chinese in Beijing, China; HWE, Hardy-Weinberg equilibrium; NA, not available.

MAF (Allele) and MAF (%) in CHB (NCBI GRCh38.p2 assembly) were downloaded from the online database of dbSNP website (HAPMAP-CHB; http://www.ncbi.nlm.nih.gov/projects/SNP/index.html).

The excluded SNPs were those with strong LD (D′ > 0.8). According to the LD results, we then re-selected these SNPs for calculation of the cumulative effect. The re-selected SNPs included 13 SNPs in the 13 closest genes: rs1046934 in *TSEN15*, rs3791679 in *EFEMP1*, rs3771381 in *ZNF638*, rs10935120 in *CEP63*, rs7632381 in *ZBTB38*, rs13131350 in *LCORL*, rs6845999 in *HHIP*, rs4240326 in *ANAPC10*, rs6470764 in *GSDMC*, rs12338076 in *QSOX2*, rs4842838 in *ADAMTSL3*, rs258324 in *CDK10*, and rs4308051 in *CABLES1*. The association was statistically evaluated by the odds ratio (OR) and its corresponding 95% confidence interval (CI) in the additive genetic model. As shown in Table 2, these 13 genetic SNPs showed a statistically significant association with FSS risk (*p*-value < 0.00005).

**Table 2:** Association between human height related genetic SNPs and familial short stature occurrence.

rs ID	Gene	Chr.	Risk allele	Homozygous dominant/Heterozygous/Homozygous recessive genotype frequency		Additive model	
				Familial short stature cases (N = 978)	Controls (N = 1129)	OR (95%CI)	*P*
rs1046934	*TSEN15*	1	A	287/500/189	270/548/311	1.32 (1.16–1.49)	1.06E-05
rs3791679	*EFEMP1*	2	C	629/311/34	625/443/59	1.39 (1.19–1.61)	1.96E-05
rs3771381	*ZNF638*	2	A	278/495/205	416/530/183	1.31 (1.16–1.48)	1.83E-05
rs10935120	*CEP63*	3	A	623/317/38	816/285/26	1.43 (1.22–1.68)	1.27E-05
rs7632381	*ZBTB38*	3	T	479/424/75	459/536/134	1.35 (1.18–1.54)	1.17E-05
rs13131350	*LCORL*	4	G	432/433/112	639/424/66	1.55 (1.36–1.78)	2.06E-10
rs6845999	*HHIP*	4	C	639/306/32	637/425/66	1.41 (1.22–1.64)	6.86E-06
rs4240326	*ANAPC10*	4	G	571/350/57	557/466/106	1.37 (1.19–1.59)	5.95E-06
rs6470764	*GSDMC*	8	T	590/336/51	593/435/101	1.35 (1.18–1.54)	2.57E-05
rs12338076	*QSOX2*	9	A	564/330/83	536/475/117	1.33 (1.16–1.52)	2.92E-05
rs4842838	*ADAMTSL3*	15	G	484/400/94	652/424/51	1.42 (1.23–1.63)	8.51E-07
rs258324	*CDK10*	16	C	528/388/62	510/505/114	1.37 (1.19–1.56)	7.32E-06
rs4308051	*CABLES1*	18	T	636/304/38	831/272/26	1.43 (1.22–1.69)	1.39E-05

Abbreviations: SNP, single nucleotide polymorphism; Chr., chromosome; FSS, familial short stature; OR, odds ratio; CI, confidence interval.

OR calculation was conducted according to the defined risk alleles.

### Cumulative effects of the 13 human height genetic SNPs on FSS risk

In accordance with the potential additive inheritance model for the individual SNP association analysis (Table 2), we subdivided the three genotypes of each SNP into three groups: risk allele homozygote (the risk genotype is coded as “2”), risk allele heterozygote (the risk genotype is coded as “1”), and Non-risk allele homozygote (the non-risk genotype is coded as “0”) (Table S6, Figures S2 and S3). We calculated the multi-locus genetic risk score (GRS) for each individual by summing the number of risk alleles (0/1/2) for each of the 13 SNPs weighted by their estimated effect sizes (log OR) (Table S6 and Figure S3). Estimates of the association between the weighted GRS divided into quartiles (Q1–Q4) and FSS were calculated using a logistic regression model; individuals in genetic risk score Q1 served as the reference. As shown in Table 3, there was a continuous increase in FSS risk with the cumulative weighted GRS quartile numbers (*p* < 0.001; Cochran-Armitage trend test). That is, compared with individuals in Q1, those identified in Q2, Q3, and Q4 had a significant association with increased FSS risk, with an increasing risk for each quartile (Table 3). These results suggested a cumulative effect of these 13 SNPs on FSS risk.

**Table 3:** Association between genetic risk score from the 13 human height genetic loci and FSS risk

Weighted genetic risk score (wGRS) quartile	FSS (%)	Controls (%)	OR	95% CI	*P* value
	N = 978	N = 1129			
	N (%)	N (%)			
Q1	144 (14.7)	412 (36.5)	Ref.	Ref.	Ref.
Q2	199 (20.3)	282 (25.0)	2.02	1.55–2.63	<***0***.***001***
Q3	254 (26.0)	278 (24.6)	2.61	2.03–3.37	<***0***.***001***
Q4	381 (39.0)	157 (13.9)	6.94	5.32–9.06	<***0***.***001***
Cochran-Armitage Trend Test					<***0***.***001***

Abbreviations: FSS, familial short stature; OR, odds ratio; CI, confidence interval; Ref., reference.

The 13 human height genetic loci and the respective risk genotypes were shown in Table S3.

The controls were the individuals whose body height was 75% above the general population in Taiwan.

Weighted genetic risk score quartile 1 (Q1): 0 < wGRS <= 11.88; Weighted genetic risk score quartile 2 (Q2): 11.88 < wGRS <= 13.50; Weighted genetic risk score quartile 3 (Q3): 13.50 < wGRS <= 15.05; Weighted genetic risk score quartile 4 (Q4): 15.05 < wGRS.

### Functional analysis

We applied these 13 SNPs re-selected from the LD results to possible pathway mapping and further functional validation to explore possible functional relations among the genes affected by these 13 SNPs. The network analysis identified a single cluster of 35 genes that included 11 associated genes discovered in this study (Figure S4) related to functions of embryonic development, organismal development, and tissue development. Six of these genes are all located in the nucleus: *ZBTB38*, *ZNF638*, *LCORL*, *CABLES1*, *CDK10*, and *TSEN15*.

## Discussion

Human height GWAS have led to the discovery of thousands of novel genetic variants^14–24^. In this work, we initiated our FSS study on a single ethnic group (Han Chinese) and investigated the associations of FSS risk with genetic variants previously related to human height in GWAS, both alone and cumulatively. We identified a continuous increase in FSS risk with increasing genetic risk score quartiles, suggesting a cumulative effect of these 13 SNPs on FSS risk. These data suggest that FSS represents a suitable research object for investigating short stature genetics. To our knowledge, this is the first study reporting the genetic profile of FSS.

The LD analysis revealed several SNPs in strong LD that have been linked to adult height in previous genetic studies, mapping to genes such as *COLGALT2* and *TSEN15*, *EFEMP1* (implicated in bone and cartilage development pathways, including the constitutions of the extracellular matrix), *ZBTB38*, *LCORL*, *HHIP*, *ANAPC10*, *GSDMC*, *QSOX2*, *ADAMTSL3*, and *CABLES1* ^14, 16–20, 25^. Other SNPs were found to be independent, located near the *ZNF638* and *CEP63* genes. CEP63 recruits the cell cycle regulatory protein CDK1 to the centrosome, and has been associated with regulation of mitotic entry, centrosome amplification, and genome maintenance, thereby affecting cell proliferation. Only one SNP was identified for chromosome 16, related to *CDK10*.

Network cluster analysis revealed functions of these 13 genes that are mainly related to embryonic development, organismal development, and tissue development. Two of the genes found to be associated with FSS belong to the zinc finger protein family: *ZBTB38* and *ZNF638*. There were seven relevant SNPs in *ZBTB38*: rs6440003, rs6763931, rs724016, rs7632381, rs1344672, rs9825379, and rs10513137 that have previously been reported in human height GWAS^12, 16, 18, 19, 25^. Moreover, genetic variants in *ZBTB38*, including rs724016, rs1582874, rs11919556, rs6440006, rs7612543, rs62282002, and rs18651435, have also been implicated in idiopathic short stature (ISS)^29^. *ZBTB38* encodes a zinc finger- and BTB domain-containing protein 38. It belongs to the family of zinc finger proteins and thus serves as a zinc finger transcriptional activator that binds methylated DNA; such potential suppression of the transcription of methylated regions might affect adult stature by regulating the production of insulin-like growth factor-II^30^. Therefore, ZBTB38 may affect adult height, ISS, and FSS by regulating the protein expression of IGF-II. In addition, *ZBTB*38 also regulates the transcription of tyrosine hydroxylase, the rate-limiting enzyme in catecholamine synthesis^31^.

Moreover, the SNP rs3771381 in *ZNF638*, encoding zinc finger protein 68, was also associated with FSS risk. *ZNF638* genetic variants have been reported in human height GWAS^25, 32^. ZNF638 is a nucleoplasmic protein that binds to cytidine-rich sequences in double-stranded DNA^33^. This zinc finger protein is a member of the large class of transcription factors that participates in skeletal development^34^ and regulates adipocyte differentiation^35^. *ZNF638* has been reported to be a transcriptional coregulator of the early regulator of adipogenesis via induction of PPARγ in cooperation with CCAAT/enhancer binding proteins (C/EBPs)^35, 36^.

*LCORL* encodes a ligand-dependent nuclear receptor corepressor-like protein and is a transcription factor. Polymorphisms in this gene are associated with measures of skeletal frame size and adult height in human and in livestock animals^24, 25, 37–40^. SNPs affecting the transcription or translation of *LCORL* may result in up- or down-regulation of the expression of genes involved in growth^38^, and may affect bone formation, skeletal frame size, as well as body height; thus, further functional investigations of these variants are warranted. One SNP (rs13131350) within this gene was associated with FSS in our study. This SNP was also reported in a meta-analysis study of GWAS of adult height in East Asians^25^. Regulation of *LCORL* protein expression may affect bone formation, skeletal frame size, as well as body height; thus, further functional investigations are warranted.

There were two cell cycle regulation-related genes—*CABLES1* and *CDK10*—associated with FSS in our study. According to the present study, four SNPs in *CABLES1* are associated with FSS in Han Chinese in Taiwan: rs4800452, rs4369779, rs4308051, and rs8094261, which have also been reported in human height GWAS^14, 18, 23, 25^. *CABLES1* encodes a Cdk5 and Abl enzyme substrate protein 1, and serves as a candidate tumor suppressor that negatively regulates cell growth by inhibiting cyclin-dependent kinases^41, 42^. Loss of *CABLES1* protein expression has been observed at high frequency in human colorectal, lung, ovarian, and endometrial hyperplasia and in endometrial cancers^42–45^. CABLES1 protein overexpression induces apoptosis and inhibits cell growth by stabilizing p21 and decreasing cyclin-dependent kinase 2 kinase activity, thereby affecting cell proliferation^46^.

The other cell cycle-regulatory gene is *CDK10*. We found one SNP (rs258324) located in *CDK10* associated with FSS, which was also reported in a human height GWAS^25^. *CDK10* encodes cyclin-dependent kinase 10 and belongs to the CDK subfamily of the family of Ser/Thr protein kinases, which are necessary for cell cycle progression. *CDK10* plays important roles in cellular proliferation and in regulation of the G2-M phase of the cell cycle^47, 48^. Therefore, *CABLES1* and *CDK10* may affect adult height and FSS by regulating cell survival, cell cycle, and cellular proliferation.

We identified one SNP (rs1046934) in *TSEN15* associated with FSS. This SNP has been reported in a human height GWAS study^14^. *TSEN15* encodes a highly conserved subunit of a tRNA-splicing endonuclease that catalyzes the removal of introns from tRNA precursors^49, 50^. tRNA splicing is a fundamental process for cell growth and division and is highly conserved among vertebrates. Mutations in *TSEN15* cause neurogenetic disorders, including pontocerebellar hypoplasia and progressive microcephaly^50–52^. These findings are suggestive of *TSEN15*′s importance in brain development. However, the roles of *TSEN15* in adult height and FSS remain unclear and further functional characterization is needed.

In conclusion, our study suggests that 13 SNPs related to human height (according to previous GWAS) are associated with FSS risk alone and cumulatively. This is the first report on the genetic profile of FSS. Such information may be helpful for further research on short stature in the Han Chinese population, and for future functional studies dealing with growth and development. Confirmatory studies with a larger sample size and with functional characterization of these candidate genes would be warranted in the near future.

## Methods

### Study participants

The case population consisted of 978 individuals with FSS sequentially enrolled from the Children’s Hospital, China Medical University, Taichung, Taiwan, from August 1999 to September 2014. All participants were unrelated Han Chinese children who were residents of central Taiwan and fulfilled the diagnostic criteria of FSS^26, 28^. The criteria for FSS in our association study were (1) height less than the 3^rd^ percentile of the corresponding population, (2) normal annual growth rate, (3) bone age appropriate for chronologic age, (4) a family history of short stature, (5) normal onset of puberty, and (6) normal results of physical examination. The controls consisted of 1,129 subjects who were randomly selected from the Taiwan Biobank (http://www.twbiobank.org.tw/new_web/index.php). The criteria for controls for inclusion in our association study were (1) no history of FSS diagnosis, (2) body height above that of the top 75th percentile of the general population in Taiwan; and (3) age <61 years. All the participating FSS cases and controls were of Han Chinese origin, which constitutes 98% of the Taiwanese population. The ethnic background was assigned in accordance with the results of self-reported questionnaires. All research was performed in accordance with the relevant guidelines and regulations. The study protocol was approved by the institutional review board and the ethics committee of Human Studies Committee of China Medical University Hospital. Written informed consent was obtained from the participants, their parent, or legal guardian in accordance with the institutional requirements and the Declaration of Helsinki principles.

### SNP selection, genotyping, and quality control

We initially included genetic SNPs listed as supplementary files from previous human height GWAS^14–24^. The genetic resources for the human height GWAS are shown in Table S7. The repeated SNPs were removed and the resulting 1,033 SNPs from 14 GWAS of human height are listed in Table S1. Genotyping of these 1,033 SNPs was performed in both cases and controls. Genomic DNA was extracted from peripheral blood leukocytes according to standard protocols (Qiagen Genomic DNA Isolation Kit; Qiagen, Taichung, Taiwan). The DNA concentration and quality were confirmed on a NanoDrop 1000 spectrophotometer (Thermo Fisher Scientific, Taichung, Taiwan). SNPs of each sample were genotyped using a custom-designed VeraCode GoldenGate Genotyping Assay System (Illumina; http://www.illumina.com/)^53–55^. Genotypic data were quality controlled, and SNPs were excluded from further analysis if (1) the MAF was less than 5% in the controls (the Han Chinese population); (2) the total call rate was less than 95% for both cases and controls; or (3) the SNP significantly departed from Hardy-Weinberg equilibrium proportions for the controls (*p* < 0.05). Then SNPs that passed the correction test were considered to be significant (*P* = 0.05/1,033 = 0.00005).

### Statistical analysis

Hardy-Weinberg equilibrium for genotype distributions of controls in this study was evaluated by the goodness-of-fit χ^2^ test (Table 1).

Alleles were identified by genotyping and direct counting. The difference of allelic frequency in the additive model between the cases and controls were measured by ORs with 95% CIs using logistical regression models (Table 2). Deviation from the additive model was also tested for 34 selected SNPs and a *p*-value for the DOMDEV test <1.47 × 10^−3^ was considered significant (*p* = 0.05/34) (Table S2). All statistical analyses were performed in the SPSS software, v12.0 for Windows (IBM, Armonk, NY, USA).

For the analysis of haplotype blocks, we used the Lewontin D′ and R^2^ measure to evaluate the intermarker coefficient of linkage disequilibrium in both controls and FSS cases^56^. The confidence interval for LD was estimated using a resampling procedure and was used to construct the haplotype blocks^57, 58^.

## Electronic supplementary material

Supplementary Information
