# Supplementary material for: Association of human height-related genetic variants with familial short stature in Han Chinese in Taiwan
Source: Sci Rep. 2017 Jul 25;7:6372. doi: 10.1038/s41598-017-06766-z (PMC5527114; doi:10.1038/s41598-017-06766-z)
Supplement: Supplementary file 1 — Supplementary Information [file 41598_2017_6766_MOESM1_ESM.pdf]

Association of human height-related genetic variants with familial short stature in Han Chinese in Taiwan

Ying-Ju Lin[1,2], Wen-Ling Liao[3,4], Chung-Hsing Wang[5], Li-Ping Tsai[6], Chih-Hsin Tang[7], Chien-Hsiun Chen[2,8], Jer-Yuarn Wu[2,8], Wen-Miin Liang[9], Ai-Ru Hsieh[9], Chi-Fung Cheng[9], Jin-Hua Chen[10], Wen-Kuei Chien[11], Ting-Hsu Lin[1], Chia-Ming Wu[1], Chiu-Chu Liao[1], Shao-Mei Huang[1] & Fuu-Jen Tsai[1,2,5,12,*]

[1]Genetic Center, Department of Medical Research, China Medical University Hospital, Taichung, Taiwan. [2]School of Chinese Medicine, China Medical University, Taichung, Taiwan. [3]Graduate Institute of Integrated Medicine, China Medical University, Taichung, Taiwan. [4]Center for Personalized Medicine, China Medical University Hospital, Taichung, Taiwan. [5]Children's Hospital of China Medical University, Taichung, Taiwan. [6]Department of Pediatrics, Buddhist Tzu Chi General Hospital, Taipei Branch, Taipei, Taiwan. [7]Graduate Institute of Biomedical Sciences, China Medical University, Taichung, Taiwan. [8]Institute of Biomedical Sciences, Academia Sinica, Taipei, Taiwan. [9]Graduate Institute of Biostatistics, School of Public Health, China Medical University, Taichung, Taiwan. [10]Biostatistics Center and School of Public Health, Taipei Medical University, Taipei, Taiwan. [11]National Applied Research Laboratories, National Center for High-performance Computing, Hsinchu, Taiwan. [12]Department of Biotechnology and Bioinformatics, Asia University, Taichung, Taiwan.

Ying-Ju Lin and Wen-Ling Liao contributed equally to this work.

*Correspondence and requests for materials should be addressed to Fuu-Jen Tsai, MD, PhD, Genetic Center, Department of Medical Research, China Medical University Hospital, No. 2, Yuh-Der Road, Taichung, Taiwan. Tel.: +886 4-22052121 ext. 2041; Fax: +886 4-22033295. E-mail: d0704@mail.cmuh.org.tw

You created this PDF from an application that is not licensed to print to novaPDF printer (http://www.novapdf.com)

Table S1 | Reference for genetic loci identified from GWAS of human height

	rs ID	Gene	Chr.	Position	Reference	Year	PMID number	Population
1	rs425277	*PRKCZ*	1	2069172	Wood AR et al., 2014	2014	25282103	**European**
2	rs9434723	*H6PD*	1	9292282	Wood AR et al., 2014	2014	25282103	**European**
3	rs10779751	*FRAP1*	1	11284336	Wood AR et al., 2014	2014	25282103	**European**
4	rs2284746	*MFAP2*	1	17306675	He M et al., 2015	2015	25429064	**European**
5	rs3738814	*ATP13A2*	1	17331676	He M et al., 2015	2015	25429064	**Asian**
6	rs12137162	*CAPZB*	1	19763396	Wood AR et al., 2014	2014	25282103	**European**
7	rs212524	*ECE1*	1	21583311	Wood AR et al., 2014	2014	25282103	**European**
8	rs3767141	*HSPG2*	1	22216279	Soranzo N et al., 2009	2009	19343178	**European**
9	rs1014987	*WNT4*	1	22498824	Wood AR et al., 2014	2014	25282103	**European**
10	rs2806561	*LUZP1*	1	23504795	Wood AR et al., 2014	2014	25282103	**European**
11	rs1738475	*HTR1D*	1	23536891	Lango Allen H et al., 2010	2010	20881960	**European**
12	rs4601530	*CLIC4*	1	25044111	Wood AR et al., 2014	2014	25282103	**European**
13	rs926438	*TMEM57*	1	25753638	Chan Y et al., 2015	2015	25865494	**Various**
14	rs17163588	*PDIK1L*	1	26450009	Wood AR et al., 2014	2014	25282103	**European**
15	rs7532866	*LIN28*	1	26741544	Lango Allen H et al., 2010	2010	20881960	**European**
16	rs2219320	*HMGN2*	1	26803430	Wood AR et al., 2014	2014	25282103	**European**
17	rs12119525	*SLC9A1*	1	27503662	Wood AR et al., 2014	2014	25282103	**European**
18	rs209918	*COL9A2*	1	40777842	Wood AR et al., 2014	2014	25282103	**European**
19	rs6686842	*SCMH1*	1	41530871	Weedon MN et al., 2008	2008	18391952	**European**
20	rs6600365	*SCMH1*	1	41556253	Wood AR et al., 2014	2014	25282103	**European**
21	rs2154319	*SCMH1*	1	41745770	Lango Allen H et al., 2010	2010	20881960	**European**
22	rs3014219	*AKR1A1*	1	46024454	Wood AR et al., 2014	2014	25282103	**European**
23	rs564914	*FOXD2*	1	47915233	Wood AR et al., 2014	2014	25282103	**European**
24	rs12855	*CDKN2C*	1	51440093	Wood AR et al., 2014	2014	25282103	**European**
25	rs3013749	*GLIS1*	1	54072759	Cho YS et al., 2009	2009	19396169	**Korean**

You created this PDF from an application that is not licensed to print to novaPDF printer (http://www.novapdf.com)

26	rs2815379	*SLC35D1*	1	67510474	Wood AR et al., 2014	2014	25282103	**European**
27	rs6699417	*PKN2*	1	89123443	Lango Allen H et al., 2010	2010	20881960	**European**
28	rs7551732	*PKN2*	1	89139041	Wood AR et al., 2014	2014	25282103	**European**
29	rs12145922	*PKN2*	1	89146234	Lanktree MB et al., 2011	2011	21194676	**European**
30	rs17113369	*RWDD3*	1	95787223	Wood AR et al., 2014	2014	25282103	**European**
31	rs12047268		1	103473494	Yang J et al., 2012	2012	22426310	**European**
32	rs7517682	*COL11A1*	1	103519589	Wood AR et al., 2014	2014	25282103	**European**
33	rs4338381	*COL11A1*	1	103572927	He M et al., 2015	2015	25429064	**East Asian**
34	rs12120956	*CAPZA1*	1	113202571	Wood AR et al., 2014	2014	25282103	**European**
35	rs1321666	*WDR3*	1	118492052	Wood AR et al., 2014	2014	25282103	**European**
36	rs7513464	*SPAG17*	1	118849762	He M et al., 2015	2015	25429064	**European**
37	rs9428104	*SPAG17*	1	118855587	Wood AR et al., 2014	2014	25282103	**European**
38	rs17038182		1	118868405	Yang J et al., 2012	2012	22426310	**European**
39	rs12735613	*SPAG17*	1	118883973	Weedon MN et al., 2008	2008	18391952	**European**
40	rs1409156	*TBX15*	1	119491784	Chan Y et al., 2015	2015	25865494	**Various**
41	rs12144094	*PHGDH*	1	120264823	Wood AR et al., 2014	2014	25282103	**European**
42	rs2120003		1	146690635	Yang J et al., 2012	2012	22426310	**European**
43	rs6658763	*FMO5*	1	146692373	Wood AR et al., 2014	2014	25282103	**European**
44	rs11205277	*SV2A*	1	149892872	He M et al., 2015	2015	25429064	**European**
45	rs3767627	*OTUD7B*	1	149938898	Wood AR et al., 2014	2014	25282103	**European**
46	rs956796	*ANP32E*	1	150186091	Chan Y et al., 2015	2015	25865494	**Various**
47	rs2298265	*ZNF687*	1	151259043	Wood AR et al., 2014	2014	25282103	**European**
48	rs12086448		1	160393905	Yang J et al., 2012	2012	22426310	**European**
49	rs6688100	*VANGL2*	1	160399586	Wood AR et al., 2014	2014	25282103	**European**
50	rs4656220	*PRRX1*	1	170649277	Wood AR et al., 2014	2014	25282103	**European**
51	rs17346452	*DNM3*	1	172053287	Lango Allen H et al., 2010	2010	20881960	**European**
52	rs6694089	*DNM3*	1	172083881	Wood AR et al., 2014	2014	25282103	**European**
53	rs12125882	*DNM3*	1	172141403	Wood AR et al., 2014	2014	25282103	**European**

You created this PDF from an application that is not licensed to print to novaPDF printer (http://www.novapdf.com)

54	rs678962	*DNM3*	1	172189889	Gudbjartsson DF et al., 2008	2008	18391951	**European**
55	rs12410416	*DNM3*	1	172193820	He M et al., 2015	2015	25429064	**European**
56	rs2421992	*DNM3*	1	172241251	Wood AR et al., 2014	2014	25282103	**European**
57	rs1325598	*PAPPA2*	1	176792249	He M et al., 2015	2015	25429064	**European**
58	rs1325596	*PAPPA2*	1	176794066	Wood AR et al., 2014	2014	25282103	**European**
59	rs9425569		1	182942202	Yang J et al., 2012	2012	22426310	**European**
60	rs4652773	*LAMC1*	1	183054827	Wood AR et al., 2014	2014	25282103	**European**
61	rs756199	*GLT25D2*	1	184002874	Okada Y et al., 2010	2010	20189936	**Japanese**
62	rs3814333	*GLT25D2*	1	184007119	Wood AR et al., 2014	2014	25282103	**European**
63	rs1926872	*GLT25D2*	1	184018475	He M et al., 2015	2015	25429064	**European**
64	rs2274432	*C1orf19,GLT25D2*	1	184020945	Gudbjartsson DF et al., 2008	2008	18391951	**European**
65	rs1046934	*TSEN15*	1	184023529	Lango Allen H et al., 2010	2010	20881960	**European**
66	rs2275325	*ZC3H11A*	1	203800735	Wood AR et al., 2014	2014	25282103	**European**
67	rs10863936	*DTL*	1	212237798	Wood AR et al., 2014	2014	25282103	**European**
68	rs4472734	*PTPN14*	1	214618185	He M et al., 2015	2015	25429064	**East Asian**
69	rs6540834	*PTPN14*	1	214627419	Wood AR et al., 2014	2014	25282103	**European**
70	rs1244981	*KCNK2*	1	215046892	Wood AR et al., 2014	2014	25282103	**European**
71	rs10495098	*TGFB2*	1	218516310	Wood AR et al., 2014	2014	25282103	**European**
72	rs1890995	*TGFB2*	1	218604678	He M et al., 2015	2015	25429064	**European**
73	rs6684205	*TGFB2*	1	218609702	Lango Allen H et al., 2010	2010	20881960	**European**
74	rs900	*TGFB2*	1	218614905	Lanktree MB et al., 2011	2011	21194676	**European**
75	rs991967	*TGFB2*	1	218615451	Wood AR et al., 2014	2014	25282103	**European**
76	rs12411277	*TGFB2*	1	218975475	Wood AR et al., 2014	2014	25282103	**European**
77	rs11118171	*LYPLAL1*	1	219047869	Yang J et al., 2012	2012	22426310	**European**
78	rs4428898	*SLC30A10*	1	219739966	Wood AR et al., 2014	2014	25282103	**European**
79	rs11118346	*LYPLAL1*	1	219743719	Lango Allen H et al., 2010	2010	20881960	**European**
80	rs1935157	*HLX*	1	221317258	Wood AR et al., 2014	2014	25282103	**European**

You created this PDF from an application that is not licensed to print to novaPDF printer (http://www.novapdf.com)

81	rs6696239	*ZNF678*	1	227750068	Wood AR et al., 2014	2014	25282103	**European**
82	rs1390401	*ZNF678*	1	227797950	Weedon MN et al., 2008	2008	18391952	**European**
83	rs12081818		1	227820374	Yang J et al., 2012	2012	22426310	**European**
84	rs10799445	*JMJD4*	1	227911883	Lango Allen H et al., 2010	2010	20881960	**European**
85	rs10048625	*MYT1L*	2	1775648	Wood AR et al., 2014	2014	25282103	**European**
86	rs3885668	*KLF11*	2	10178479	Wood AR et al., 2014	2014	25282103	**European**
87	rs2345835	*RDH14*	2	18574952	Wood AR et al., 2014	2014	25282103	**European**
88	rs7601531		2	19967944	Yang J et al., 2012	2012	22426310	**European**
89	rs13006748	*WDR35*	2	20151819	Wood AR et al., 2014	2014	25282103	**European**
90	rs6731333		2	24113354	Yang J et al., 2012	2012	22426310	**European**
91	rs7561273	*LOC388931*	2	24247514	Wood AR et al., 2014	2014	25282103	**European**
92	rs2278483	*CENPO*	2	25040082	Wood AR et al., 2014	2014	25282103	**European**
93	rs4665736	*RBJ*	2	25187599	He M et al., 2015	2015	25429064	**European**
94	rs1866146	*POMC*	2	25380573	Lanktree MB et al., 2011	2011	21194676	**European**
95	rs2289195	*DNMT3A*	2	25463483	Wood AR et al., 2014	2014	25282103	**European**
96	rs11694842	*DNMT3A*	2	25482970	He M et al., 2015	2015	25429064	**European**
97	rs10460566	*DNMT3A*	2	25483121	Chan Y et al., 2015	2015	25865494	**Various**
98	rs780094	*GCKR*	2	27741237	Wood AR et al., 2014	2014	25282103	**European**
99	rs6751657	*LTBP1*	2	33405151	Wood AR et al., 2014	2014	25282103	**European**
100	rs3769528	*LTBP1*	2	33471192	He M et al., 2015	2015	25429064	**European**
101	rs41464348	*LTBP1*	2	33527299	Kim JJ et al., 2010	2010	19893584	**Korean**
102	rs3755206	*CRIM1*	2	36683428	He M et al., 2015	2015	25429064	**East Asian**
103	rs711245	*CRIM1*	2	36768875	Wood AR et al., 2014	2014	25282103	**European**
104	rs6544089	*CDC42EP3*	2	37758745	Wood AR et al., 2014	2014	25282103	**European**
105	rs7606245	*SLC8A1*	2	42018118	He M et al., 2015	2015	25429064	**East Asian**
106	rs9309101	*THADA*	2	43629612	Wood AR et al., 2014	2014	25282103	**European**
107	rs2341459	*C2orf34*	2	44768202	Lango Allen H et al., 2010	2010	20881960	**European**
108	rs7596521	*SOCS5*	2	46918665	Soranzo N et al., 2009	2009	19343178	**European**
109	rs12474201	*SOCS5*	2	46921285	Wood AR et al., 2014	2014	25282103	**European**

You created this PDF from an application that is not licensed to print to novaPDF printer (http://www.novapdf.com)

110	rs17822294		2	46960004	Yang J et al., 2012	2012	22426310	**European**
111	rs354196	*SPTBN1*	2	54966407	Wood AR et al., 2014	2014	25282103	**European**
112	rs4146922	*PNPT1*	2	56067182	He M et al., 2015	2015	25429064	**European**
113	rs1367226	*EFEMP1*	2	56089540	Chan Y et al., 2015	2015	25865494	**Various**
114	rs3791679	*EFEMP1*	2	56096892	Wood AR et al., 2014	2014	25282103	**European**
115	rs3791675	*EFEMP1*	2	56111309	He M et al., 2015	2015	25429064	**European, East Asian**
116	rs1822469	*PPP3R1*	2	68454685	Lanktree MB et al., 2011	2011	21194676	**European**
117	rs2120335	*PPP3R1*	2	68495002	Wood AR et al., 2014	2014	25282103	**European**
118	rs3771381	*ZNF638*	2	71560665	He M et al., 2015	2015	25429064	**East Asian**
119	rs7568069	*ZNF638*	2	71584485	Wood AR et al., 2014	2014	25282103	**European**
120	rs867529	*EIF2AK3*	2	88913273	He M et al., 2015	2015	25429064	**European**
121	rs11684404	*EIF2AK3*	2	88924622	Wood AR et al., 2014	2014	25282103	**European**
122	rs13388725	*GCC2*	2	109047190	Wood AR et al., 2014	2014	25282103	**European**
123	rs2166898	*GLI2*	2	121612659	Wood AR et al., 2014	2014	25282103	**European**
124	rs7567288	*NAP5*	2	134434824	Wood AR et al., 2014	2014	25282103	**European**
125	rs4953951	*ZRANB3*	2	136187345	Wood AR et al., 2014	2014	25282103	**European**
126	rs749234	*ZEB2*	2	145231349	Wood AR et al., 2014	2014	25282103	**European**
127	rs540652	*NOSTRIN*	2	169707428	Wood AR et al., 2014	2014	25282103	**European**
128	rs12987566	*METTL8*	2	172152646	Wood AR et al., 2014	2014	25282103	**European**
129	rs6746356	*SP3*	2	174815898	Wood AR et al., 2014	2014	25282103	**European**
130	rs7567851	*PDE11A*	2	178684720	Wood AR et al., 2014	2014	25282103	**European**
131	rs12693589	*STAT1*	2	191832662	Wood AR et al., 2014	2014	25282103	**European**
132	rs6435143	*NOP5/NOP58*	2	203194256	Wood AR et al., 2014	2014	25282103	**European**
133	rs4425077	*FN1*	2	216410516	Wood AR et al., 2014	2014	25282103	**European**
134	rs12329133	*TNP1*	2	217935116	Chan Y et al., 2015	2015	25865494	**Various**
135	rs1351164	*TNS1*	2	218271898	Lango Allen H et al., 2010	2010	20881960	**European**
136	rs994533	*TNS1*	2	218284278	Wood AR et al., 2014	2014	25282103	**European**
137	rs992157	*PNKD/TMBIM1*	2	219154781	Wood AR et al., 2014	2014	25282103	**European**
138	rs2305833	*VIL1*	2	219305404	Wood AR et al., 2014	2014	25282103	**European**

You created this PDF from an application that is not licensed to print to novaPDF printer (http://www.novapdf.com)

139	rs611203	*PLCD4*	2	219472325	He M et al., 2015	2015	25429064	**European**
140	rs1541777	*TTLL4*	2	219587291	Yang J et al., 2012	2012	22426310	**European**
141	rs4674354	*CCDC108*	2	219903723	Chan Y et al., 2015	2015	25865494	**Various**
142	rs6741325	*CCDC108*	2	219907699	Yang J et al., 2012	2012	22426310	**European**
143	rs12470505	*CCDC108*	2	219908369	Wood AR et al., 2014	2014	25282103	**European**
144	rs1052483	*IHH, CRYBA2, FEV, SLC23A3, TUBA1*	2	219934348	Gudbjartsson DF et al., 2008	2008	18391951	**European**
145	rs6724465	*IHH*	2	219943846	Weedon MN et al., 2008	2008	18391952	**European**
146	rs16859517	*NHEJ1*	2	219949184	Wood AR et al., 2014	2014	25282103	**European**
147	rs7588654	*NHEJ1*	2	219983030	He M et al., 2015	2015	25429064	**European**
148	rs6753739	*SLC23A3*	2	220028900	He M et al., 2015	2015	25429064	**European**
149	rs12621643	*KCNE4*	2	223917983	Chan Y et al., 2015	2015	25865494	**Various**
150	rs6761041	*SERPINE2*	2	225030129	Wood AR et al., 2014	2014	25282103	**European**
151	rs2629046	*SERPINE2*	2	225047744	Lango Allen H et al., 2010	2010	20881960	**European**
152	rs7598759	*NCL*	2	232321956	Yang J et al., 2012	2012	22426310	**European**
153	rs4973429	*C2orf52*	2	232377818	Wood AR et al., 2014	2014	25282103	**European**
154	rs2679184	*NPPC*	2	232779223	Wood AR et al., 2014	2014	25282103	**European**
155	rs2580816	*NPPC*	2	232797966	Lango Allen H et al., 2010	2010	20881960	**European**
156	rs10460436	*NPPC*	2	232815341	He M et al., 2015	2015	25429064	**European**
157	rs3116168	*DIS3L2*	2	232989831	Wood AR et al., 2014	2014	25282103	**European**
158	rs3103296	*DIS3L2*	2	233034495	Lanktree MB et al., 2011	2011	21194676	**European**
159	rs6728302	*DIS3L2*	2	233053961	He M et al., 2015	2015	25429064	**European, East Asian**
160	rs7571816	*DIS3L2*	2	233077064	Okada Y et al., 2010	2010	20189936	**Japanese**
161	rs2343240	*DIS3L2*	2	233087483	Wood AR et al., 2014	2014	25282103	**European**
162	rs6717918	*DIS3L2*	2	233155110	Okada Y et al., 2010	2010	20189936	**Japanese**
163	rs7571716	*EIF4E2*	2	233441420	Yang J et al., 2012	2012	22426310	**European**
164	rs13393800	*EIF4E2*	2	233442091	Wood AR et al., 2014	2014	25282103	**European**
165	rs4676386	*KIF1A*	2	241774986	Yang J et al., 2012	2012	22426310	**European**

You created this PDF from an application that is not licensed to print to novaPDF printer (http://www.novapdf.com)

166	rs4344931	*AGXT*	2	241818527	Wood AR et al., 2014	2014	25282103	**European**
167	rs2633761	*ITPR1*	3	4728104	Chan Y et al., 2015	2015	25865494	**Various**
168	rs6772112		3	11641535	Yang J et al., 2012	2012	22426310	**European**
169	rs13078528	*VGLL4*	3	11646954	Wood AR et al., 2014	2014	25282103	**European**
170	rs9816693	*VILL*	3	38047954	Wood AR et al., 2014	2014	25282103	**European**
171	rs3915129	*CTNNB1*	3	41243742	Wood AR et al., 2014	2014	25282103	**European**
172	rs2240919	*ITIH3*	3	52831701	Wood AR et al., 2014	2014	25282103	**European**
173	rs2336725	*RTF1*	3	53118739	Lango Allen H et al., 2010	2010	20881960	**European**
174	rs2581830	*RFT1*	3	53134098	Wood AR et al., 2014	2014	25282103	**European**
175	rs2034172	*WNT5A*	3	55411763	Wood AR et al., 2014	2014	25282103	**European**
176	rs4681933		3	56660229	Yang J et al., 2012	2012	22426310	**European**
177	rs9835332	*C3orf63*	3	56667682	Wood AR et al., 2014	2014	25282103	**European**
178	rs1098018	*FLNB*	3	57979767	Lei SF et al., 2009	2009	19039035	**European**
179	rs1718460	*FLNB*	3	58006413	Lei SF et al., 2009	2009	19039035	**European**
180	rs1658342	*FLNB*	3	58009259	Lei SF et al., 2009	2009	19039035	**European**
181	rs1658351	*FLNB*	3	58013573	Wood AR et al., 2014	2014	25282103	**European**
182	rs865726	*FLNB*	3	58018879	Lei SF et al., 2009	2009	19039035	**European**
183	rs839232	*FLNB*	3	58020826	Lei SF et al., 2009	2009	19039035	**European**
184	rs4681784	*FLNB*	3	58071644	Lei SF et al., 2009	2009	19039035	**European**
185	rs9834312	*FLNB*	3	58082709	Lei SF et al., 2009	2009	19039035	**European**
186	rs3772993	*FLNB*	3	58114884	Lei SF et al., 2009	2009	19039035	**European**
187	rs6794009	*PTPRG*	3	61513495	Wood AR et al., 2014	2014	25282103	**European**
188	rs17806888	*SUCLG2*	3	67416322	Wood AR et al., 2014	2014	25282103	**European**
189	rs2175513	*FAM19A1*	3	68622366	Wood AR et al., 2014	2014	25282103	**European**
190	rs9863706	*RYBP*	3	72437413	Lango Allen H et al., 2010	2010	20881960	**European**
191	rs12330322	*RYBP*	3	72455355	Wood AR et al., 2014	2014	25282103	**European**
192	rs13072744	*RYBP*	3	72509637	He M et al., 2015	2015	25429064	**European**
193	rs17009984		3	72637183	Chan Y et al., 2015	2015	25865494	**Various**
194	rs7633464	*DCBLD2*	3	98715823	Wood AR et al., 2014	2014	25282103	**European**

You created this PDF from an application that is not licensed to print to novaPDF printer (http://www.novapdf.com)

195	rs9825951	*COL8A1*	3	99269921	Wood AR et al., 2014	2014	25282103	**European**
196	rs1797625	*C3orf17*	3	112826415	Wood AR et al., 2014	2014	25282103	**European**
197	rs2718423		3	114208597	Yang J et al., 2012	2012	22426310	**European**
198	rs1533269	*ZBTB20*	3	114214611	Wood AR et al., 2014	2014	25282103	**European**
199	rs1546391	*ZBTB20*	3	114697457	Wood AR et al., 2014	2014	25282103	**European**
200	rs7636293	*C3orf47*	3	129045906	He M et al., 2015	2015	25429064	**European**
201	rs6439167	*C3orf47*	3	129050756	Lango Allen H et al., 2010	2010	20881960	**European**
202	rs6439168	*H1FX*	3	129050943	Wood AR et al., 2014	2014	25282103	**European**
203	rs4974480	*ANAPC13*	3	134178562	Wood AR et al., 2014	2014	25282103	**European**
204	rs10935120	*ANAPC13 o rCEP63*	3	134233092	Weedon MN et al., 2008	2008	18391952	**European**
205	rs6440003	*ZBTB38*	3	141094209	Weedon MN et al., 2008	2008	18391952	**European**
206	rs6763931	*ZBTB38*	3	141102833	He M et al., 2015	2015	25429064	**European, East Asian**
207	rs724016	*ZBTB38*	3	141105570	Wood AR et al., 2014	2014	25282103	**European**
208	rs7632381	*ZBTB38, ACPL2*	3	141106063	Kim JJ et al., 2010	2010	19893584	**Korean**
209	rs1344672	*ZBTB38, ACPL2*	3	141125705	Kim JJ et al., 2010	2010	19893584	**Korean**
210	rs9825379	*ZBTB38*	3	141137035	Okada Y et al., 2010	2010	20189936	**Japanese**
211	rs10513137	*ZBTB38, ACPL2*	3	141143430	Kim JJ et al., 2010	2010	19893584	**Korean**
212	rs936339	*PCOLCE2*	3	142535505	Wood AR et al., 2014	2014	25282103	**European**
213	rs4325879	*CCNL1*	3	156851984	Wood AR et al., 2014	2014	25282103	**European**
214	rs16828478	*SHOX2*	3	157591239	Cho YS et al., 2009	2009	19396169	**Korean**
215	rs9818941		3	157686457	Yang J et al., 2012	2012	22426310	**European**
216	rs6441170	*SHOX2*	3	157806960	Wood AR et al., 2014	2014	25282103	**European**
217	rs4345115	*GOLIM4, SERPINI1*	3	167837748	Gudbjartsson DF et al., 2008	2008	18391951	**European**
218	rs2421649		3	169197333	Chan Y et al., 2015	2015	25865494	**Various**
219	rs7652177	*FNDC3B*	3	171969077	Wood AR et al., 2014	2014	25282103	**European**
220	rs4243400	*FNDC3B*	3	171970859	He M et al., 2015	2015	25429064	**European**
221	rs509035	*GHSR*	3	172163449	Wood AR et al., 2014	2014	25282103	**European**

You created this PDF from an application that is not licensed to print to novaPDF printer (http://www.novapdf.com)

222	rs572169	*GHSR*	3	172165727	He M et al., 2015	2015	25429064	**European**
223	rs9858528	*KLHL24*	3	183355405	Wood AR et al., 2014	2014	25282103	**European**
224	rs6784185	*IGF2BP2*	3	185473065	Yang J et al., 2012	2012	22426310	**European**
225	rs16860216	*IGF2BP2*	3	185488882	Chan Y et al., 2015	2015	25865494	**Various**
226	rs720390	*IGF2BP2*	3	185548683	Wood AR et al., 2014	2014	25282103	**European**
227	rs2300921	*SFRS10*	3	185651001	Wood AR et al., 2014	2014	25282103	**European**
228	rs4686904	*BCL6*	3	187438522	Wood AR et al., 2014	2014	25282103	**European**
229	rs7646824	*OSTN*	3	190815978	Wood AR et al., 2014	2014	25282103	**European**
230	rs9841435	*CCDC50*	3	191111160	Wood AR et al., 2014	2014	25282103	**European**
231	rs3958122	*SLBP*	4	1693931	Wood AR et al., 2014	2014	25282103	**European**
232	rs2247341	*SLBP/FGFR3*	4	1701317	Lango Allen H et al., 2010	2010	20881960	**European**
233	rs867245	*POLN*	4	2218888	Wood AR et al., 2014	2014	25282103	**European**
234	rs2916448	*LYAR*	4	4276918	Lettre G et al., 2008	2008	18391950	**European**
235	rs868489	*MGC21874*	4	7055253	Wood AR et al., 2014	2014	25282103	**European**
236	rs6829680	*AFAP1*	4	7912333	Wood AR et al., 2014	2014	25282103	**European**
237	rs2302580	*CPZ*	4	8608634	Wood AR et al., 2014	2014	25282103	**European**
238	rs763318	*RAB28*	4	12963574	Wood AR et al., 2014	2014	25282103	**European**
239	rs7678436	*NCAPG-LCORL*	4	17797966	Okada Y et al., 2010	2010	20189936	**Japanese**
240	rs16895802	*NCAPG*	4	17815889	He M et al., 2015	2015	25429064	**European, East Asian**
241	rs6842303	*LCORL, NCAPG*	4	17854055	Gudbjartsson DF et al., 2008	2008	18391951	**European**
242	rs6854334	*LCORL*	4	17861210	Soranzo N et al., 2009	2009	19343178	**European**
243	rs6817306	*LCORL*	4	17868058	Soranzo N et al., 2009	2009	19343178	**European**
244	rs13131350	*LCORL*	4	17877487	He M et al., 2015	2015	25429064	**European, East Asian**
245	rs7692995	*LCORL*	4	17936634	Wood AR et al., 2014	2014	25282103	**European**
246	rs16896068	*LCORL*	4	17944840	Weedon MN et al., 2008	2008	18391952	**European**
247	rs961014	*LCORL*	4	18010384	Chan Y et al., 2015	2015	25865494	**Various**
248	rs16896276	*LCORL*	4	18015156	Yang J et al., 2012	2012	22426310	**European**

You created this PDF from an application that is not licensed to print to novaPDF printer (http://www.novapdf.com)

249	rs6830062	*LCORL, NCAPG*	4	18017730	Gudbjartsson DF et al., 2008	2008	18391951	**European**
250	rs2011603	*NCAPG,LCORL*	4	18025484	Cho YS et al., 2009	2009	19396169	**Korean**
251	rs6449353	*LCORL*	4	18033488	Lango Allen H et al., 2010	2010	20881960	**European**
252	rs16994718	*KLF3*	4	38688362	Wood AR et al., 2014	2014	25282103	**European**
253	rs11096991	*RFC1*	4	39320631	Lanktree MB et al., 2011	2011	21194676	**European**
254	rs2306596	*RFC1*	4	39343940	Wood AR et al., 2014	2014	25282103	**European**
255	rs1996422	*FRYL*	4	48687351	Wood AR et al., 2014	2014	25282103	**European**
256	rs13113518	*CLOCK*	4	56399648	Wood AR et al., 2014	2014	25282103	**European**
257	rs4864546	*CLOCK*	4	56404127	Lanktree MB et al., 2011	2011	21194676	**European**
258	rs3796529	*REST*	4	57797414	Lanktree MB et al., 2011	2011	21194676	**European**
259	rs2227901	*REST*	4	57798189	He M et al., 2015	2015	25429064	**European**
260	rs17081935	*C4orf14*	4	57823476	Wood AR et al., 2014	2014	25282103	**European**
261	rs3733309	*POLR2B*	4	57857188	He M et al., 2015	2015	25429064	**European**
262	rs9993613	*ADAMTS3*	4	73476014	Wood AR et al., 2014	2014	25282103	**European**
263	rs7697556	*ADAMTS3*	4	73515313	Lango Allen H et al., 2010	2010	20881960	**European**
264	rs16848425	*ADAMTS3*	4	73515825	He M et al., 2015	2015	25429064	**European**
265	rs710841	*PRKG2*	4	82149831	Soranzo N et al., 2009	2009	19343178	**European**
266	rs1662845	*PRKG2*	4	82154282	Lettre G et al., 2008	2008	18391950	**European**
267	rs7661369		4	82166066	Yang J et al., 2012	2012	22426310	**European**
268	rs2011962	*RASGEF1B*	4	82220324	He M et al., 2015	2015	25429064	**European**
269	rs6813055	*DMP1*	4	88630031	Chan Y et al., 2015	2015	25865494	**Various**
270	rs13136331		4	88707081	Chan Y et al., 2015	2015	25865494	**Various**
271	rs10010325	*TET2*	4	106106353	Lango Allen H et al., 2010	2010	20881960	**European**
272	rs2454206	*TET2*	4	106196951	He M et al., 2015	2015	25429064	**European**
273	rs12639764	*TET2*	4	106216205	Wood AR et al., 2014	2014	25282103	**European**
274	rs2101975		4	106216667	Yang J et al., 2012	2012	22426310	**European**
275	rs7659107	*CAMK2D*	4	114742249	Wood AR et al., 2014	2014	25282103	**European**
276	rs7659604	*BBS7*	4	122665514	Lanktree MB et al., 2011	2011	21194676	**European**

You created this PDF from an application that is not licensed to print to novaPDF printer (http://www.novapdf.com)

277	rs6838153	*EXOSC9*	4	122720999	Wood AR et al., 2014	2014	25282103	**European**
278	rs6824258		4	122769967	Yang J et al., 2012	2012	22426310	**European**
279	rs12513181	*NUDT6*	4	123835656	Wood AR et al., 2014	2014	25282103	**European**
280	rs17016123	*INPP4B*	4	143355338	Cho YS et al., 2009	2009	19396169	**Korean**
281	rs11100790	*SMARCA5*	4	144442611	Wood AR et al., 2014	2014	25282103	**European**
282	rs7654571	*HHIP*	4	145321006	Wood AR et al., 2014	2014	25282103	**European**
283	rs17720281	*HHIP*	4	145543776	Yang J et al., 2012	2012	22426310	**European**
284	rs6845999	*HHIP*	4	145565826	Wood AR et al., 2014	2014	25282103	**European**
285	rs7689420	*HHIP*	4	145568352	Lango Allen H et al., 2010	2010	20881960	**European**
286	rs1812175	*HHIP*	4	145574844	Wood AR et al., 2014	2014	25282103	**European**
287	rs6854783	*HHIP*	4	145643079	Weedon MN et al., 2008	2008	18391952	**European**
288	rs1492820	*HHIP*	4	145650021	Lettre G et al., 2008	2008	18391950	**European**
289	rs4240326	*ANAPC10*	4	145839264	Wood AR et al., 2014	2014	25282103	**European**
290	rs6823268	*ANAPC10*	4	145982563	He M et al., 2015	2015	25429064	**European**
291	rs13150868	*ESSPL*	4	152180671	Chan Y et al., 2015	2015	25865494	**Various**
292	rs955748	*WWC2*	4	184215675	Wood AR et al., 2014	2014	25282103	**European**
293	rs1450822		5	4520856	Lettre G et al., 2008	2008	18391950	**European**
294	rs17410035	*C5orf22*	5	31541142	Wood AR et al., 2014	2014	25282103	**European**
295	rs1173735	*NPR3*	5	32771379	Yang J et al., 2012	2012	22426310	**European**
296	rs1173736	*NPR3*	5	32771938	Lanktree MB et al., 2011	2011	21194676	**European**
297	rs3811958	*NPR3*	5	32772043	Wood AR et al., 2014	2014	25282103	**European**
298	rs9292468	*C5orf23*	5	32819073	Wood AR et al., 2014	2014	25282103	**European**
299	rs13183624	*C5orf23*	5	32821168	Chan Y et al., 2015	2015	25865494	**Various**
300	rs1173727	*NPR3*	5	32830521	Lango Allen H et al., 2010	2010	20881960	**European**
301	rs10472828	*NPR3*	5	32888818	Soranzo N et al., 2009	2009	19343178	**European**
302	rs11745439	*TARS*	5	33230034	Wood AR et al., 2014	2014	25282103	**European**
303	rs1004202	*UGT3A2*	5	36065463	Chan Y et al., 2015	2015	25865494	**Various**
304	rs301901	*NIPBL*	5	37046626	Wood AR et al., 2014	2014	25282103	**European**
305	rs3812040	*DAB2*	5	39426020	Wood AR et al., 2014	2014	25282103	**European**

You created this PDF from an application that is not licensed to print to novaPDF printer (http://www.novapdf.com)

306	rs6180	*GHR*	5	42719239	He M et al., 2015	2015	25429064	**East Asian**
307	rs2961830	*ISL1*	5	50454732	Wood AR et al., 2014	2014	25282103	**European**
308	rs7704138	*SLC38A9*	5	54944262	He M et al., 2015	2015	25429064	**European**
309	rs7716219	*SLC38A9*	5	54955071	Wood AR et al., 2014	2014	25282103	**European**
310	rs11958779	*SLC38A9*	5	55001899	Lango Allen H et al., 2010	2010	20881960	**European**
311	rs162089	*DDX4*	5	55118675	Cho YS et al., 2009	2009	19396169	**Korean**
312	rs2662027	*MIER3*	5	56254485	Wood AR et al., 2014	2014	25282103	**European**
313	rs9291926	*PIK3R1*	5	67599656	Wood AR et al., 2014	2014	25282103	**European**
314	rs820848	*HEXB*	5	73964660	Wood AR et al., 2014	2014	25282103	**European**
315	rs12519505	*AP3B1*	5	77505876	Wood AR et al., 2014	2014	25282103	**European**
316	rs7712162	*PAPD4*	5	78945171	Wood AR et al., 2014	2014	25282103	**European**
317	rs32855	*FAM151B*	5	79836192	Wood AR et al., 2014	2014	25282103	**European**
318	rs6894139	*MEF2C*	5	88327782	Wood AR et al., 2014	2014	25282103	**European**
319	rs10037512	*MEF2C*	5	88354675	He M et al., 2015	2015	25429064	**European**
320	rs2247870	*GPR98*	5	90151589	Wood AR et al., 2014	2014	25282103	**European**
321	rs12186664	*PCSK1*	5	95630225	Wood AR et al., 2014	2014	25282103	**European**
322	rs6594336	*FER*	5	108073085	Chan Y et al., 2015	2015	25865494	**Various**
323	rs1582931	*CCDC100*	5	122657199	Wood AR et al., 2014	2014	25282103	**European**
324	rs7708474	*CEP120*	5	122676525	He M et al., 2015	2015	25429064	**European**
325	rs6887276	*SLC12A2*	5	127378294	Wood AR et al., 2014	2014	25282103	**European**
326	rs26024	*FBN2*	5	127696022	Wood AR et al., 2014	2014	25282103	**European**
327	rs274546	*SLC22A5*	5	131699867	Lango Allen H et al., 2010	2010	20881960	**European**
328	rs6596075	*LOC441108*	5	131742228	Chan Y et al., 2015	2015	25865494	**Various**
329	rs537930		5	134348703	Yang J et al., 2012	2012	22426310	**European**
330	rs526896	*PITX1*	5	134356705	Wood AR et al., 2014	2014	25282103	**European**
331	rs31198	*PITX1, PCBD2, CATSPER3 TXNDC15,DDX 46, CAMLG*	5	134372685	Gudbjartsson DF et al., 2008	2008	18391951	**European**

You created this PDF from an application that is not licensed to print to novaPDF printer (http://www.novapdf.com)

332	rs4624820	*SPRY4*	5	141681788	Wood AR et al., 2014	2014	25282103	**European**
333	rs2974438	*SLIT3*	5	168250903	Wood AR et al., 2014	2014	25282103	**European**
334	rs4282339	*SLIT3*	5	168256240	Lango Allen H et al., 2010	2010	20881960	**European**
335	rs4620037	*FGF18*	5	170875097	Wood AR et al., 2014	2014	25282103	**European**
336	rs1529701	*FGF18*	5	171000977	Wood AR et al., 2014	2014	25282103	**European**
337	rs33852	*FBXW11*	5	171189571	Wood AR et al., 2014	2014	25282103	**European**
338	rs12153391	*FBXW11*	5	171203438	Wood AR et al., 2014	2014	25282103	**European**
339	rs4868126	*FBXW11*	5	171283469	Wood AR et al., 2014	2014	25282103	**European**
340	rs1368380	*FBXW11*	5	171285632	Wood AR et al., 2014	2014	25282103	**European**
341	rs17075869	*STC2*	5	172811280	Chan Y et al., 2015	2015	25865494	**Various**
342	rs6885032		5	172983279	Chan Y et al., 2015	2015	25865494	**Various**
343	rs889014	*STC2-BOD1*	5	172984114	He M et al., 2015	2015	25429064	**European**
344	rs7733195	*FAM44B*	5	172994624	Wood AR et al., 2014	2014	25282103	**European**
345	rs6556079		5	172997078	Yang J et al., 2012	2012	22426310	**European**
346	rs4868645	*RNF44*	5	175947118	Chan Y et al., 2015	2015	25865494	**Various**
347	rs6556301		5	176527577	Chan Y et al., 2015	2015	25865494	**Various**
348	rs11750568	*ADAMTS2*	5	178535713	Wood AR et al., 2014	2014	25282103	**European**
349	rs6879260	*GFPT2*	5	179731014	Wood AR et al., 2014	2014	25282103	**European**
350	rs932445	*GMDS*	6	2167225	Wood AR et al., 2014	2014	25282103	**European**
351	rs163071	*GMDS*	6	2193062	Chan Y et al., 2015	2015	25865494	**Various**
352	rs17603945	*RREB1*	6	7213016	Wood AR et al., 2014	2014	25282103	**European**
353	rs2714357	*RREB1*	6	7225995	Soranzo N et al., 2009	2009	19343178	**European**
354	rs12198986	*BMP6*	6	7720059	Gudbjartsson DF et al., 2008	2008	18391951	**European**
355	rs3812163	*BMP6*	6	7725760	Lango Allen H et al., 2010	2010	20881960	**European**
356	rs9328445	*BMP6*	6	7792947	Chan Y et al., 2015	2015	25865494	**Various**
357	rs9405356	*BMP6*	6	7804377	Chan Y et al., 2015	2015	25865494	**Various**
358	rs742106	*DTNBP1*	6	15524480	Soranzo N et al., 2009	2009	19343178	**European**

You created this PDF from an application that is not licensed to print to novaPDF printer (http://www.novapdf.com)

359	rs12199222	*NUP153, CAP2, KIF13A*	6	17699322	Gudbjartsson DF et al., 2008	2008	18391951	**European**
360	rs6921309		6	18618735	Cho YS et al., 2009	2009	19396169	**Korean**
361	rs1865760	*SLC17A2*	6	25916979	He M et al., 2015	2015	25429064	**East Asian**
362	rs9393681	*TRIM38,HIST1 H1A*	6	26008260	Cho YS et al., 2009	2009	19396169	**Korean**
363	rs4141885	*HIST1H1E*	6	26157481	Wood AR et al., 2014	2014	25282103	**European**
364	rs806794	*HIST1H2BF*	6	26200677	He M et al., 2015	2015	25429064	**European**
365	rs10946808	*Histone class 1,Butyrophilin genes*	6	26233387	Gudbjartsson DF et al., 2008	2008	18391951	**European**
366	rs9358913	*HIST1H4F*	6	26239404	Soranzo N et al., 2009	2009	19343178	**European**
367	rs1233627	*TRIM27*	6	28751727	Wood AR et al., 2014	2014	25282103	**European**
368	rs3129109	*OR2J3*	6	29084232	Lango Allen H et al., 2010	2010	20881960	**European**
369	rs11970475	*UBD*	6	29526377	He M et al., 2015	2015	25429064	**East Asian**
370	rs9404952	*HLA-G*	6	29804165	Wood AR et al., 2014	2014	25282103	**European**
371	rs2517538	*LOC729792*	6	31013541	Cho YS et al., 2009	2009	19396169	**Korean**
372	rs2251830	*HCG22*	6	31016978	He M et al., 2015	2015	25429064	**East Asian**
373	rs2233969	*C6orf15,PSORS 1C1*	6	31080432	Cho YS et al., 2009	2009	19396169	**Korean**
374	rs3823418	*PSORS1C1*	6	31100942	He M et al., 2015	2015	25429064	**East Asian**
375	rs1265097	*PSORS1C1/PSO RS1C2*	6	31106459	Wood AR et al., 2014	2014	25282103	**European**
376	rs6457374	*HLA-C*	6	31272261	Wood AR et al., 2014	2014	25282103	**European**
377	rs2596494	*HLA-B*	6	31323838	Lanktree MB et al., 2011	2011	21194676	**European**
378	rs13437082	*HLA-B*	6	31354560	Soranzo N et al., 2009	2009	19343178	**European**
379	rs4711269	*HLA-B*	6	31354819	Soranzo N et al., 2009	2009	19343178	**European**
380	rs2256183	*MICA*	6	31380529	Lango Allen H et al., 2010	2010	20881960	**European**
381	rs2516448	*MICA*	6	31390410	Lanktree MB et al., 2011	2011	21194676	**European**

You created this PDF from an application that is not licensed to print to novaPDF printer (http://www.novapdf.com)

382	rs2844479	*HLA class III*	6	31572956	Gudbjartsson DF et al., 2008	2008	18391951	**European**
383	rs2857693	*BAT2*	6	31588384	Wood AR et al., 2014	2014	25282103	**European**
384	rs2077102		6	31611840	Kim JJ et al., 2010	2010	19893584	**Korean**
385	rs589428		6	31848220	Chan Y et al., 2015	2015	25865494	**Various**
386	rs185819	*HLA class III*	6	32050067	Gudbjartsson DF et al., 2008	2008	18391951	**European**
387	rs1061807		6	32136838	Yang J et al., 2012	2012	22426310	**European**
388	rs6457620	*HLA locus*	6	32663999	Lango Allen H et al., 2010	2010	20881960	**European**
389	rs3129254	*COL11A2*	6	33108287	Wood AR et al., 2014	2014	25282103	**European**
390	rs7742369	*HMGA1*	6	34165721	Okada Y et al., 2010	2010	20189936	**Japanese**
391	rs12214804	*HMGA1*	6	34188866	Wood AR et al., 2014	2014	25282103	**European**
392	rs1776897	*HMGA1*	6	34195011	He M et al., 2015	2015	25429064	**European, East Asian**
393	rs2780226	*HMGA1*	6	34199092	Lango Allen H et al., 2010	2010	20881960	**European**
394	rs1150781	*HMGA1*	6	34214322	Lanktree MB et al., 2011	2011	21194676	**European**
395	rs6918981	*NUDT3*	6	34238514	He M et al., 2015	2015	25429064	**Asian**
396	rs3734254	*PPARD*	6	35395010	Lanktree MB et al., 2011	2011	21194676	**European**
397	rs4713858	*ANKS1, TCP11, ZNF76, DEF6,SCUBE3*	6	35402785	Gudbjartsson DF et al., 2008	2008	18391951	**European**
398	rs4713902	*FKBP5*	6	35614026	Wood AR et al., 2014	2014	25282103	**European**
399	rs16895130	*CCND3*	6	41924931	Wood AR et al., 2014	2014	25282103	**European**
400	rs9472414	*SUPT3H/RUNX2*	6	44946506	Lango Allen H et al., 2010	2010	20881960	**European**
401	rs9296450		6	44953786	Yang J et al., 2012	2012	22426310	**European**
402	rs10948197	*SUPT3H*	6	44967490	He M et al., 2015	2015	25429064	**East Asian**
403	rs9395066	*SUPT3H, RUNX2*	6	45095163	Gudbjartsson DF et al., 2008	2008	18391951	**European**
404	rs10948222	*SUPT3H*	6	45244415	Wood AR et al., 2014	2014	25282103	**European**

You created this PDF from an application that is not licensed to print to novaPDF printer (http://www.novapdf.com)

405	rs9395264	*CD2AP*	6	47475022	Wood AR et al., 2014	2014	25282103	**European**
406	rs12190423	*OGFRL1*	6	72202711	Wood AR et al., 2014	2014	25282103	**European**
407	rs12209223	*FILIP1*	6	76164589	Wood AR et al., 2014	2014	25282103	**European**
408	rs6903448	*FILIP1*	6	76173832	Wood AR et al., 2014	2014	25282103	**European**
409	rs9360921	*SENP6*	6	76265642	Lango Allen H et al., 2010	2010	20881960	**European**
410	rs6931421		6	80880138	Chan Y et al., 2015	2015	25865494	**Various**
411	rs9341808		6	80953257	Chan Y et al., 2015	2015	25865494	**Various**
412	rs648831	*BCKDHB*	6	80956208	Wood AR et al., 2014	2014	25282103	**European**
413	rs1341278	*BCKDHB*	6	81038921	Wood AR et al., 2014	2014	25282103	**European**
414	rs9443804	*BCKDHB*	6	81315597	Wood AR et al., 2014	2014	25282103	**European**
415	rs310421	*FAM46A*	6	81792063	Wood AR et al., 2014	2014	25282103	**European**
416	rs310405	*FAM46A*	6	81800362	Lango Allen H et al., 2010	2010	20881960	**European**
417	rs310402	*FAM46A*	6	81800492	Yang J et al., 2012	2012	22426310	**European**
418	rs3828760	*FAM46A*	6	82456984	Wood AR et al., 2014	2014	25282103	**European**
419	rs761391	*TBX18*	6	85448103	Wood AR et al., 2014	2014	25282103	**European**
420	rs7759938	*LIN28B*	6	105378954	Lango Allen H et al., 2010	2010	20881960	**European**
421	rs314263	*LIN28B*	6	105392745	Wood AR et al., 2014	2014	25282103	**European**
422	rs314277	*LIN28B*	6	105407662	Lettre G et al., 2008	2008	18391950	**European**
423	rs314268	*LIN28B, HACE1, BVES, POPDC3*	6	105417978	Gudbjartsson DF et al., 2008	2008	18391951	**European**
424	rs479744	*FOXO3*	6	109020032	Wood AR et al., 2014	2014	25282103	**European**
425	rs6920372	*PPIL6*	6	109723939	Wood AR et al., 2014	2014	25282103	**European**
426	rs9487094	*PPIL6, CD164, SMPD2,MNICAL1,ZBTB24*	6	109742015	Gudbjartsson DF et al., 2008	2008	18391951	**European**
427	rs1476387	*ZBTB24*	6	109764535	Lanktree MB et al., 2011	2011	21194676	**European**
428	rs1046943	*ZBTB24*	6	109783941	Lango Allen H et al., 2010	2010	20881960	**European**
429	rs3734652		6	109786980	Yang J et al., 2012	2012	22426310	**European**

You created this PDF from an application that is not licensed to print to novaPDF printer (http://www.novapdf.com)

430	rs1405212	*VGLL2*	6	117490664	Wood AR et al., 2014	2014	25282103	**European**
431	rs961764	*VGLL2*	6	117522156	Lango Allen H et al., 2010	2010	20881960	**European**
432	rs389663	*DCBLD1*	6	117868051	Wood AR et al., 2014	2014	25282103	**European**
433	rs4895801	*NCOA7*	6	126216403	Wood AR et al., 2014	2014	25282103	**European**
434	rs1415701	*L3MBTL3*	6	130345835	He M et al., 2015	2015	25429064	**European**
435	rs6569648	*L3MBTL3*	6	130349119	Lango Allen H et al., 2010	2010	20881960	**European**
436	rs6899976	*L3MBTL3, SAMD3*	6	130358428	Gudbjartsson DF et al., 2008	2008	18391951	**European**
437	rs7740107	*L3MBTL3*	6	130374461	Wood AR et al., 2014	2014	25282103	**European**
438	rs6921207	*EPB41L2*	6	131327956	Wood AR et al., 2014	2014	25282103	**European**
439	rs6570507	*GPR126*	6	142679572	Soranzo N et al., 2009	2009	19343178	**European**
440	rs4896582	*GPR126*	6	142703877	Wood AR et al., 2014	2014	25282103	**European**
441	rs3748069	*GPR126*	6	142767633	Gudbjartsson DF et al., 2008	2008	18391951	**European**
442	rs7763064	*GPR126*	6	142797289	Lango Allen H et al., 2010	2010	20881960	**European**
443	rs262115		6	142817407	Yang J et al., 2012	2012	22426310	**European**
444	rs6911389	*PHACTR2*	6	144079629	Chan Y et al., 2015	2015	25865494	**Various**
445	rs2748483	*GRM1*	6	146335560	Wood AR et al., 2014	2014	25282103	**European**
446	rs543650	*ESR1*	6	152110943	Lango Allen H et al., 2010	2010	20881960	**European**
447	rs488133	*ESR1*	6	152125444	Lanktree MB et al., 2011	2011	21194676	**European**
448	rs6902771	*ESR1*	6	152157881	Wood AR et al., 2014	2014	25282103	**European**
449	rs3020418	*ESR1*	6	152345162	Wood AR et al., 2014	2014	25282103	**European**
450	rs1832871	*TULP4*	6	158722034	Wood AR et al., 2014	2014	25282103	**European**
451	rs486359	*SLC22A3*	6	160774441	Chan Y et al., 2015	2015	25865494	**Various**
452	rs991946	*T*	6	166329862	Wood AR et al., 2014	2014	25282103	**European**
453	rs9459531		6	166333799	Yang J et al., 2012	2012	22426310	**European**
454	rs7774834	*THBS2*	6	169349731	Wood AR et al., 2014	2014	25282103	**European**
455	rs798544	*GNA12*	7	2763102	Gudbjartsson DF et al., 2008	2008	18391951	**European**

You created this PDF from an application that is not licensed to print to novaPDF printer (http://www.novapdf.com)

456	rs798497	*GNA12*	7	2795957	Wood AR et al., 2014	2014	25282103	**European**
457	rs7777484	*GNA12*	7	2814271	He M et al., 2015	2015	25429064	**European**
458	rs1182188	*GNA12*	7	2869985	Soranzo N et al., 2009	2009	19343178	**European**
459	rs1182179	*GNA12*	7	2873648	Soranzo N et al., 2009	2009	19343178	**European**
460	rs4725061	*GLCCI1*	7	8086639	Wood AR et al., 2014	2014	25282103	**European**
461	rs929637	*TMEM106B*	7	12276522	Wood AR et al., 2014	2014	25282103	**European**
462	rs1523632	*AGR2*	7	17069968	Soranzo N et al., 2009	2009	19343178	**European**
463	rs4470914	*TWISTNB*	7	19616522	Lango Allen H et al., 2010	2010	20881960	**European**
464	rs2390151	*TWISTNB*	7	19642100	Wood AR et al., 2014	2014	25282103	**European**
465	rs3807931	*ITGB8*	7	20381674	Wood AR et al., 2014	2014	25282103	**European**
466	rs12538581	*ITGB8*	7	20399117	Yang J et al., 2012	2012	22426310	**European**
467	rs1175000	*CDCA7L*	7	22024040	Okada Y et al., 2010	2010	20189936	**Japanese**
468	rs12538407	*IGF2BP3*	7	23521316	Wood AR et al., 2014	2014	25282103	**European**
469	rs1055144	*NFE2L3*	7	25871109	Wood AR et al., 2014	2014	25282103	**European**
470	rs864745	*JAZF1*	7	28180556	He M et al., 2015	2015	25429064	**East Asian**
471	rs849141	*JAZF1*	7	28185091	Soranzo N et al., 2009	2009	19343178	**European**
472	rs1635852	*JAZF1*	7	28189411	Okada Y et al., 2010	2010	20189936	**Japanese**
473	rs1708299	*JAZF1*	7	28189946	Lango Allen H et al., 2010	2010	20881960	**European**
474	rs537124		7	28203142	Yang J et al., 2012	2012	22426310	**European**
475	rs6462432	*KBTBD2*	7	32935524	Wood AR et al., 2014	2014	25282103	**European**
476	rs6974574	*STARD3NL*	7	38110073	Wood AR et al., 2014	2014	25282103	**European**
477	rs6959212	*STARD3NL*	7	38128326	Lango Allen H et al., 2010	2010	20881960	**European**
478	rs2949837		7	45994378	Chan Y et al., 2015	2015	25865494	**Various**
479	rs1007358	*IGFBP3*	7	46201355	Wood AR et al., 2014	2014	25282103	**European**
480	rs12534698		7	46408264	Yang J et al., 2012	2012	22426310	**European**
481	rs6949739	*IGFBP3*	7	46417403	Wood AR et al., 2014	2014	25282103	**European**
482	rs12540874	*GRB10*	7	50664922	Lettre G et al., 2008	2008	18391950	**European**
483	rs2715094	*GRB10*	7	50730452	Wood AR et al., 2014	2014	25282103	**European**
484	rs1113765	*SEPT14*	7	55889334	Wood AR et al., 2014	2014	25282103	**European**

You created this PDF from an application that is not licensed to print to novaPDF printer (http://www.novapdf.com)

485	rs12669267	*WBSCR28*	7	73304636	Wood AR et al., 2014	2014	25282103	**European**
486	rs17807185	*RSBN1L*	7	77308295	Wood AR et al., 2014	2014	25282103	**European**
487	rs4272	*CDK6*	7	92236829	Lanktree MB et al., 2011	2011	21194676	**European**
488	rs42039	*CDK6*	7	92244422	Wood AR et al., 2014	2014	25282103	**European**
489	rs42235	*CDK6*	7	92248076	Lango Allen H et al., 2010	2010	20881960	**European**
490	rs2040494	*CDK6*	7	92256905	Lettre G et al., 2008	2008	18391950	**European**
491	rs2282978	*CDK6, PEX1, GATAD1, ERVWE1*	7	92264410	Gudbjartsson DF et al., 2008	2008	18391951	**European**
492	rs2282979	*CDK6*	7	92264993	Okada Y et al., 2010	2010	20189936	**Japanese**
493	rs11765954	*CDK6, PEX1, GATAD1, ERVWE1*	7	92280695	Gudbjartsson DF et al., 2008	2008	18391951	**European**
494	rs6971575	*SLC25A13*	7	96039648	Wood AR et al., 2014	2014	25282103	**European**
495	rs6952113	*C7orf58*	7	120777619	Wood AR et al., 2014	2014	25282103	**European**
496	rs6962887	*CNOT4*	7	135045786	Wood AR et al., 2014	2014	25282103	**European**
497	rs3812265	*CNOT4*	7	135048804	Lanktree MB et al., 2011	2011	21194676	**European**
498	rs273945	*CREB3L2*	7	137611566	Wood AR et al., 2014	2014	25282103	**European**
499	rs822552		7	148650634	Yang J et al., 2012	2012	22426310	**European**
500	rs6955948	*TMEM176A*	7	150508720	Wood AR et al., 2014	2014	25282103	**European**
501	rs2110001	*TMEM176A*	7	150517022	Lango Allen H et al., 2010	2010	20881960	**European**
502	rs2730245	*WDR60*	7	158724789	Lettre G et al., 2008	2008	18391950	**European**
503	rs4875421	*CSMD1*	8	4827332	Wood AR et al., 2014	2014	25282103	**European**
504	rs330938		8	9017979	Chan Y et al., 2015	2015	25865494	**Various**
505	rs7823327	*PEBP4*	8	22562352	Wood AR et al., 2014	2014	25282103	**European**
506	rs4273857	*LOXL2*	8	23173053	Wood AR et al., 2014	2014	25282103	**European**
507	rs17088184	*SLC25A37*	8	23375235	Wood AR et al., 2014	2014	25282103	**European**
508	rs2013265	*ADAM28*	8	24092500	Wood AR et al., 2014	2014	25282103	**European**
509	rs3812423	*KCTD9*	8	25298710	Wood AR et al., 2014	2014	25282103	**European**

You created this PDF from an application that is not licensed to print to novaPDF printer (http://www.novapdf.com)

510	rs568610	*SCARA3*	8	27527995	Wood AR et al., 2014	2014	25282103	**European**
511	rs10448080	*EXTL3*	8	28604791	He M et al., 2015	2015	25429064	**East Asian**
512	rs6988484	*EFCAB1*	8	49413780	Wood AR et al., 2014	2014	25282103	**European**
513	rs10958476	*PLAG1*	8	57095808	Wood AR et al., 2014	2014	25282103	**European**
514	rs7833986	*PLAG1*	8	57100149	Okada Y et al., 2010	2010	20189936	**Japanese**
515	rs13273123	*PLAG1*	8	57100791	He M et al., 2015	2015	25429064	**European, East Asian**
516	rs9650315	*CHCHD7*	8	57155598	Wood AR et al., 2014	2014	25282103	**European**
517	rs7829319		8	57172232	Kim JJ et al., 2010	2010	19893584	**Korean**
518	rs7815788	*PLAG1*	8	57179020	Soranzo N et al., 2009	2009	19343178	**European**
519	rs7460090	*SDR16C5*	8	57194163	Lango Allen H et al., 2010	2010	20881960	**European**
520	rs7815909	*CHCHD7-RDHE2*	8	57200362	He M et al., 2015	2015	25429064	**European**
521	rs4738736		8	59836001	Yang J et al., 2012	2012	22426310	**European**
522	rs2956605	*CRISPLD1*	8	75883054	Wood AR et al., 2014	2014	25282103	**European**
523	rs16939034	*CRISPLD1*	8	76040583	Chan Y et al., 2015	2015	25865494	**Various**
524	rs4735677	*PXMP3*	8	78148191	Wood AR et al., 2014	2014	25282103	**European**
525	rs7846385	*PXMP3, ZFHX4*	8	78160179	Gudbjartsson DF et al., 2008	2008	18391951	**European**
526	rs6473015	*PEX2*	8	78178485	Lango Allen H et al., 2010	2010	20881960	**European**
527	rs7812578		8	82679057	Chan Y et al., 2015	2015	25865494	**Various**
528	rs7007200	*TMEM74*	8	109784938	Wood AR et al., 2014	2014	25282103	**European**
529	rs2737220	*TRPS1*	8	116637685	Wood AR et al., 2014	2014	25282103	**European**
530	rs1550162	*EIF3H*	8	117563532	Wood AR et al., 2014	2014	25282103	**European**
531	rs11989122	*EXT1*	8	118827839	He M et al., 2015	2015	25429064	**East Asian**
532	rs1599473	*NOV*	8	120475358	Wood AR et al., 2014	2014	25282103	**European**
533	rs16892729		8	120558078	Yang J et al., 2012	2012	22426310	**European**
534	rs11779459	*ZHX2*	8	123980551	Chan Y et al., 2015	2015	25865494	**Various**
535	rs8180991	*TRIB1*	8	126500350	Wood AR et al., 2014	2014	25282103	**European**
536	rs4733789	*MYC*	8	128834403	He M et al., 2015	2015	25429064	**East Asian**

You created this PDF from an application that is not licensed to print to novaPDF printer (http://www.novapdf.com)

537	rs4733724	*MLZE*	8	130723728	Wood AR et al., 2014	2014	25282103	**European**
538	rs6470764	*GSDMC*	8	130725665	Lango Allen H et al., 2010	2010	20881960	**European**
539	rs2062078	*GSDMC*	8	130734461	He M et al., 2015	2015	25429064	**European**
540	rs894343	*ZFAT*	8	135612595	Chan Y et al., 2015	2015	25865494	**Various**
541	rs11785144		8	135616199	Yang J et al., 2012	2012	22426310	**European**
542	rs12680655	*ZFAT*	8	135637337	Lango Allen H et al., 2010	2010	20881960	**European**
543	rs1036821	*ZFAT*	8	135650483	Wood AR et al., 2014	2014	25282103	**European**
544	rs6577717	*ZFAT*	8	135653832	Chan Y et al., 2015	2015	25865494	**Various**
545	rs17772163	*ZFAT*	8	135655246	Cho YS et al., 2009	2009	19396169	**Korean**
546	rs7033940	*UHRF2*	9	6440419	Wood AR et al., 2014	2014	25282103	**European**
547	rs10961780	*FREM1*	9	14898161	He M et al., 2015	2015	25429064	**East Asian**
548	rs7864648		9	16368732	Yang J et al., 2012	2012	22426310	**European**
549	rs2149163	*BNC2*	9	16455833	Wood AR et al., 2014	2014	25282103	**European**
550	rs3927536	*BNC2*	9	16787670	Wood AR et al., 2014	2014	25282103	**European**
551	rs10962832	*CNTLN*	9	17048990	Wood AR et al., 2014	2014	25282103	**European**
552	rs1576900	*ADAMTSL1*	9	18629792	Wood AR et al., 2014	2014	25282103	**European**
553	rs7871764	*WDR40A*	9	34071541	Soranzo N et al., 2009	2009	19343178	**European**
554	rs3763631	*NPR2/SPAG8*	9	35808334	Wood AR et al., 2014	2014	25282103	**European**
555	rs10972628	*OR2S2*	9	35937611	Chan Y et al., 2015	2015	25865494	**Various**
556	rs181338	*ZCCHC6*	9	89108161	Wood AR et al., 2014	2014	25282103	**European**
557	rs2814828	*SPIN1, CCRK*	9	90811182	Gudbjartsson DF et al., 2008	2008	18391951	**European**
558	rs2778031	*SPIN1*	9	90835726	Lango Allen H et al., 2010	2010	20881960	**European**
559	rs4877418		9	90836498	Yang J et al., 2012	2012	22426310	**European**
560	rs10780910	*SPIN1*	9	90849255	Wood AR et al., 2014	2014	25282103	**European**
561	rs1571892	*NFIL3*	9	94258836	Chan Y et al., 2015	2015	25865494	**Various**
562	rs7043114	*IPPK*	9	95387983	Wood AR et al., 2014	2014	25282103	**European**
563	rs9969804	*IPPK*	9	95429120	Lango Allen H et al., 2010	2010	20881960	**European**
564	rs16910061	*FBP2*	9	97314741	He M et al., 2015	2015	25429064	**East Asian**

You created this PDF from an application that is not licensed to print to novaPDF printer (http://www.novapdf.com)

565	rs558990	*FBP2*	9	97331187	He M et al., 2015	2015	25429064	**East Asian**
566	rs600130	*FBP2*	9	97338996	He M et al., 2015	2015	25429064	**East Asian**
567	rs532027	*FBP2*	9	97340038	He M et al., 2015	2015	25429064	**East Asian**
568	rs12347744	*C9orf3*	9	97575273	Wood AR et al., 2014	2014	25282103	**European**
569	rs10512248	*PTCH1*	9	98259703	Lanktree MB et al., 2011	2011	21194676	**European**
570	rs4448343	*PTCH1*	9	98266370	Wood AR et al., 2014	2014	25282103	**European**
571	rs10990303	*PTCH1*	9	98410405	Wood AR et al., 2014	2014	25282103	**European**
572	rs10978781	*ZNP510*	9	99508480	He M et al., 2015	2015	25429064	**East Asian**
573	rs10816533	*ZNP510*	9	99539138	He M et al., 2015	2015	25429064	**East Asian**
574	rs35334289	*ZNP510*	9	99540291	He M et al., 2015	2015	25429064	**East Asian**
575	rs10978953	*ZNP510*	9	99543801	He M et al., 2015	2015	25429064	**East Asian**
576	rs4344199	*ZNP510*	9	99549449	He M et al., 2015	2015	25429064	**East Asian**
577	rs10117921	*ZNP510*	9	99550434	He M et al., 2015	2015	25429064	**East Asian**
578	rs10118617	*ZNP510*	9	99550469	He M et al., 2015	2015	25429064	**East Asian**
579	rs10119466	*ZNP510*	9	99551625	He M et al., 2015	2015	25429064	**East Asian**
580	rs10119556	*ZNP782*	9	99551986	He M et al., 2015	2015	25429064	**East Asian**
581	rs10124911	*ZNP782*	9	99567384	He M et al., 2015	2015	25429064	**East Asian**
582	rs12236125	*ZNP782*	9	99583468	He M et al., 2015	2015	25429064	**East Asian**
583	rs7859940	*ZNP782*	9	99584880	He M et al., 2015	2015	25429064	**East Asian**
584	rs10124033	*ZNP782*	9	99614104	He M et al., 2015	2015	25429064	**East Asian**
585	rs953199	*XPA*	9	100482976	Chan Y et al., 2015	2015	25865494	**Various**
586	rs989393	*COL15A1*	9	101743336	Wood AR et al., 2014	2014	25282103	**European**
587	rs10820814	*FSD1L*	9	108304500	Wood AR et al., 2014	2014	25282103	**European**
588	rs9409082	*TMEM38B*	9	108901049	Wood AR et al., 2014	2014	25282103	**European**
589	rs902143	*ZNF462*	9	109181911	Wood AR et al., 2014	2014	25282103	**European**
590	rs2451948	*ZNF462*	9	109518208	Chan Y et al., 2015	2015	25865494	**Various**
591	rs7027110	*ZNF462*	9	109599046	Wood AR et al., 2014	2014	25282103	**European**
592	rs4743034	*ZNF462*	9	109632353	Gudbjartsson DF et al., 2008	2008	18391951	**European**

You created this PDF from an application that is not licensed to print to novaPDF printer (http://www.novapdf.com)

593	rs7032940	*PALM2-AKAP2, C9orf152*	9	112945405	He M et al., 2015	2015	25429064	**East Asian**
594	rs7036157	*PALM2-AKAP2, C9orf152*	9	112945774	He M et al., 2015	2015	25429064	**East Asian**
595	rs10816937	*PALM2-AKAP2, C9orf152*	9	112951989	He M et al., 2015	2015	25429064	**East Asian**
596	rs3739707	*LPAR1*	9	113792706	Wood AR et al., 2014	2014	25282103	**European**
597	rs1468758	*LPAR1*	9	113807082	Lango Allen H et al., 2010	2010	20881960	**European**
598	rs999599	*COL27A1*	9	117011595	Chan Y et al., 2015	2015	25865494	**Various**
599	rs10759774	*1-Dec*	9	118169080	Chan Y et al., 2015	2015	25865494	**Various**
600	rs10119624	*DEC1*	9	118305438	Wood AR et al., 2014	2014	25282103	**European**
601	rs13302480	*37226*	9	118465313	Yang J et al., 2012	2012	22426310	**European**
602	rs12344396	*PAPPA*	9	118921327	Wood AR et al., 2014	2014	25282103	**European**
603	rs751543	*PAPPA*	9	119122342	Lango Allen H et al., 2010	2010	20881960	**European**
604	rs1742829	*ASTN2*	9	119422807	Wood AR et al., 2014	2014	25282103	**European**
605	rs7466269	*FUBP3*	9	133464084	Wood AR et al., 2014	2014	25282103	**European**
606	rs7849585	*QSOX2*	9	139111870	Wood AR et al., 2014	2014	25282103	**European**
607	rs10858250	*QSOX2*	9	139119215	He M et al., 2015	2015	25429064	**European, East Asian**
608	rs12338076	*LHX3-QSOX2*	9	139121740	Okada Y et al., 2010	2010	20189936	**Japanese**
609	rs8413	*INPP5E*	9	139323311	Yang J et al., 2012	2012	22426310	**European**
610	rs3812591	*SEC16A*	9	139341612	Wood AR et al., 2014	2014	25282103	**European**
611	rs4332428	*AKR1C1*	10	4965434	Wood AR et al., 2014	2014	25282103	**European**
612	rs7909670	*CCDC3*	10	12918764	Lango Allen H et al., 2010	2010	20881960	**European**
613	rs12779328	*CCDC3*	10	12943973	Wood AR et al., 2014	2014	25282103	**European**
614	rs4350272	*ARHGAP21*	10	25056118	Wood AR et al., 2014	2014	25282103	**European**
615	rs7069985	*RAB18*	10	27890831	Wood AR et al., 2014	2014	25282103	**European**
616	rs12413361	*ZNF438*	10	31127166	He M et al., 2015	2015	25429064	**East Asian**
617	rs10995319	*PRKG1*	10	52762887	Wood AR et al., 2014	2014	25282103	**European**
618	rs10997979	*MYPN*	10	69937192	Wood AR et al., 2014	2014	25282103	**European**

You created this PDF from an application that is not licensed to print to novaPDF printer (http://www.novapdf.com)

619	rs4746769	*DNA2*	10	70196580	Wood AR et al., 2014	2014	25282103	**European**
620	rs779933		10	80918517	Yang J et al., 2012	2012	22426310	**European**
621	rs7916441		10	80925577	Chan Y et al., 2015	2015	25865494	**Various**
622	rs1815314	*ZMIZ1*	10	80928793	Wood AR et al., 2014	2014	25282103	**European**
623	rs703985	*ZMIZ1*	10	80940740	Chan Y et al., 2015	2015	25865494	**Various**
624	rs2145998	*PPIF*	10	81121696	Lango Allen H et al., 2010	2010	20881960	**European**
625	rs1923367	*ZCCHC24*	10	81132829	Wood AR et al., 2014	2014	25282103	**European**
626	rs2631676	*PCGF5*	10	93037409	Wood AR et al., 2014	2014	25282103	**European**
627	rs915506	*CCNJ*	10	97805074	Wood AR et al., 2014	2014	25282103	**European**
628	rs11599750	*CPN1*	10	101805442	Wood AR et al., 2014	2014	25282103	**European**
629	rs10883563	*FAM178A*	10	102684380	Wood AR et al., 2014	2014	25282103	**European**
630	rs7899004	*SUFU*	10	104341435	Wood AR et al., 2014	2014	25282103	**European**
631	rs11198820	*GRK5*	10	120961043	Chan Y et al., 2015	2015	25865494	**Various**
632	rs291979	*GRK5*	10	121129797	Wood AR et al., 2014	2014	25282103	**European**
633	rs4751815		10	122701505	Cho YS et al., 2009	2009	19396169	**Korean**
634	rs1614303	*FGFR2*	10	123396806	Wood AR et al., 2014	2014	25282103	**European**
635	rs6585827	*PLEKHA1*	10	124165615	Okada Y et al., 2010	2010	20189936	**Japanese**
636	rs7097701	*PLEKHA1*	10	124171857	Chan Y et al., 2015	2015	25865494	**Various**
637	rs10794175	*FAM53B*	10	126358073	Wood AR et al., 2014	2014	25282103	**European**
638	rs17152411	*ZRANB1*	10	126649516	He M et al., 2015	2015	25429064	**East Asian**
639	rs3781426	*CTBP2*	10	126703349	He M et al., 2015	2015	25429064	**East Asian**
640	rs11245515	*CTBP2*	10	126824068	Wood AR et al., 2014	2014	25282103	**European**
641	rs11244750	*FANK1*	10	127673877	Wood AR et al., 2014	2014	25282103	**European**
642	rs2272566	*PSMD13*	11	244552	Wood AR et al., 2014	2014	25282103	**European**
643	rs4320932	*INS-IGF2*	11	2171601	Wood AR et al., 2014	2014	25282103	**European**
644	rs17659078	*ASCL2*	11	2284590	Wood AR et al., 2014	2014	25282103	**European**
645	rs2075870	*KCNQ1*	11	2790019	Lanktree MB et al., 2011	2011	21194676	**European**
646	rs12288355	*SBF2*	11	10072849	Lei SF et al., 2009	2009	19039035	**European**
647	rs7119000	*SBF2*	11	10096240	Lei SF et al., 2009	2009	19039035	**European**

You created this PDF from an application that is not licensed to print to novaPDF printer (http://www.novapdf.com)

648	rs11042617	*SBF2*	11	10108484	Lei SF et al., 2009	2009	19039035	**European**
649	rs10734652	*SBF2*	11	10150212	Lei SF et al., 2009	2009	19039035	**European**
650	rs4323860	*SBF2*	11	10159883	Lei SF et al., 2009	2009	19039035	**European**
651	rs11042666	*SBF2*	11	10227393	Lei SF et al., 2009	2009	19039035	**European**
652	rs1867138	*SBF2*	11	10228728	Lei SF et al., 2009	2009	19039035	**European**
653	rs11042702	*SBF2*	11	10287988	Lei SF et al., 2009	2009	19039035	**European**
654	rs7108358	*SBF2*	11	10294840	Lei SF et al., 2009	2009	19039035	**European**
655	rs6484147	*SBF2*	11	10295103	Lei SF et al., 2009	2009	19039035	**European**
656	rs11042714	*SBF2*	11	10295651	Lei SF et al., 2009	2009	19039035	**European**
657	rs10500724	*SBF2*	11	10302016	Lei SF et al., 2009	2009	19039035	**European**
658	rs11042717	*SBF2*	11	10303939	Lei SF et al., 2009	2009	19039035	**European**
659	rs11607174	*SBF2*	11	10306028	Lei SF et al., 2009	2009	19039035	**European**
660	rs6485978	*TEAD1*	11	12678415	Wood AR et al., 2014	2014	25282103	**European**
661	rs7926971	*TEAD1*	11	12698040	Lango Allen H et al., 2010	2010	20881960	**European**
662	rs7937898		11	12703561	Yang J et al., 2012	2012	22426310	**European**
663	rs2033908		11	12838286	Chan Y et al., 2015	2015	25865494	**Various**
664	rs2099745	*TEAD1*	11	12924265	Wood AR et al., 2014	2014	25282103	**European**
665	rs10766065	*ARNTL*	11	13277961	Chan Y et al., 2015	2015	25865494	**Various**
666	rs2915404	*RRAS2*	11	14404825	Chan Y et al., 2015	2015	25865494	**Various**
667	rs1330	*NUCB2*	11	17316029	Lango Allen H et al., 2010	2010	20881960	**European**
668	rs757081	*NUCB2*	11	17351683	Wood AR et al., 2014	2014	25282103	**European**
669	rs11024739	*SPTY2D1*	11	18645843	He M et al., 2015	2015	25429064	**East Asian**
670	rs6483645	*DBX1*	11	20171947	Cho YS et al., 2009	2009	19396169	**Korean**
671	rs7481109		11	27298062	Chan Y et al., 2015	2015	25865494	**Various**
672	rs10767838	*C11orf46*	11	30347927	Wood AR et al., 2014	2014	25282103	**European**
673	rs3802758	*PEX16*	11	45936035	Wood AR et al., 2014	2014	25282103	**European**
674	rs1681630	*PTPRJ*	11	47969152	Wood AR et al., 2014	2014	25282103	**European**
675	rs4752805	*PTPRJ*	11	48018355	Lanktree MB et al., 2011	2011	21194676	**European**

You created this PDF from an application that is not licensed to print to novaPDF printer (http://www.novapdf.com)

676	rs10838801	*PTPRJ/SLC39A13*	11	48098280	Lango Allen H et al., 2010	2010	20881960	**European**
677	rs1814175	*FOLH1*	11	49559172	Lango Allen H et al., 2010	2010	20881960	**European**
678	rs11228763	*OR9G1,OR9G4*	11	56511160	Cho YS et al., 2009	2009	19396169	**Korean**
679	rs174547	*FADS1*	11	61570783	He M et al., 2015	2015	25429064	**East Asian**
680	rs3782089	*SSSCA1*	11	65336819	Wood AR et al., 2014	2014	25282103	**European**
681	rs4630309	*BBS1-CTSF*	11	66333072	Lanktree MB et al., 2011	2011	21194676	**European**
682	rs2510396	*GAL*	11	68417652	Wood AR et al., 2014	2014	25282103	**European**
683	rs3750972	*TPCN2*	11	68830628	Wood AR et al., 2014	2014	25282103	**European**
684	rs1938679	*CCND1*	11	69272096	He M et al., 2015	2015	25429064	**East Asian**
685	rs2509133	*TMEM16A*	11	69933717	Wood AR et al., 2014	2014	25282103	**European**
686	rs11236294	*NEU3*	11	74739934	Wood AR et al., 2014	2014	25282103	**European**
687	rs606452	*SERPINH1*	11	75276178	He M et al., 2015	2015	25429064	**European, East Asian**
688	rs634552	*SERPINH1*	11	75282052	Lango Allen H et al., 2010	2010	20881960	**European**
689	rs494459	*TREH*	11	118574675	Lango Allen H et al., 2010	2010	20881960	**European**
690	rs632124	*DDX6*	11	118613235	Wood AR et al., 2014	2014	25282103	**European**
691	rs2510897		11	118644582	Yang J et al., 2012	2012	22426310	**European**
692	rs10790381	*ARHGEF12*	11	120257495	Wood AR et al., 2014	2014	25282103	**European**
693	rs1461503	*BSX*	11	122845075	Wood AR et al., 2014	2014	25282103	**European**
694	rs11221442	*FLI1*	11	128577624	Wood AR et al., 2014	2014	25282103	**European**
695	rs654723	*FLI1*	11	128586155	Lango Allen H et al., 2010	2010	20881960	**European**
696	rs7299326	*ERC1*	12	1573005	Chan Y et al., 2015	2015	25865494	**Various**
697	rs2187642	*ETV6*	12	11855624	Gudbjartsson DF et al., 2008	2008	18391951	**European**
698	rs2856321	*ETV6*	12	11855773	Wood AR et al., 2014	2014	25282103	**European**
699	rs12228415	*ATF7IP*	12	14520701	Chan Y et al., 2015	2015	25865494	**Various**
700	rs4326884	*PDE3A*	12	20536371	Wood AR et al., 2014	2014	25282103	**European**
701	rs7137534	*PDE3A*	12	20831777	Lanktree MB et al., 2011	2011	21194676	**European**
702	rs10770705	*SLCO1C1*	12	20857467	Wood AR et al., 2014	2014	25282103	**European**

You created this PDF from an application that is not licensed to print to novaPDF printer (http://www.novapdf.com)

703	rs11047239	*SOX5*	12	24207780	Wood AR et al., 2014	2014	25282103	**European**
704	rs1861908	*KLHDC5*	12	27997409	Chan Y et al., 2015	2015	25865494	**Various**
705	rs10492364	*PTHLH*	12	28112256	Chan Y et al., 2015	2015	25865494	**Various**
706	rs2638953	*CCDC91*	12	28534415	Lango Allen H et al., 2010	2010	20881960	**European**
707	rs11049611	*CCDC91*	12	28600244	Wood AR et al., 2014	2014	25282103	**European**
708	rs12820411	*CCDC91*	12	28952342	Wood AR et al., 2014	2014	25282103	**European**
709	rs10843390	*ERGIC2*	12	29496991	Wood AR et al., 2014	2014	25282103	**European**
710	rs10880969	*SLC38A2*	12	46827023	Wood AR et al., 2014	2014	25282103	**European**
711	rs10875798		12	48732884	Chan Y et al., 2015	2015	25865494	**Various**
712	rs11170624	*ATF7*	12	54030238	He M et al., 2015	2015	25429064	**Asian**
713	rs11170631	*ATF7-ATP5G2*	12	54041192	Okada Y et al., 2010	2010	20189936	**Japanese**
714	rs1971762	*ATP5G2*	12	54058238	He M et al., 2015	2015	25429064	**Asian**
715	rs2306694	*CS*	12	56680636	Wood AR et al., 2014	2014	25282103	**European**
716	rs703830		12	56701872	Yang J et al., 2012	2012	22426310	**European**
717	rs2066808	*STAT2*	12	56737973	He M et al., 2015	2015	25429064	**European**
718	rs10877030	*CTDSP2*	12	58256714	Wood AR et al., 2014	2014	25282103	**European**
719	rs2164968	*MSRB3*	12	65677086	Wood AR et al., 2014	2014	25282103	**European**
720	rs1480474	*HMGA2*	12	66326943	Soranzo N et al., 2009	2009	19343178	**European**
721	rs1351394	*HMGA2*	12	66351826	Lango Allen H et al., 2010	2010	20881960	**European**
722	rs867633	*HMGA2*	12	66354911	Lanktree MB et al., 2011	2011	21194676	**European**
723	rs1042725	*HMGA2*	12	66358347	Weedon MN et al., 2008	2008	18391952	**European**
724	rs8756	*HMGA2*	12	66359752	Wood AR et al., 2014	2014	25282103	**European**
725	rs10748128	*FRS2*	12	69827658	Wood AR et al., 2014	2014	25282103	**European**
726	rs11177669	*LYZ, YEATS4, FRS2, CPSF6, CCT2,LRRC10*	12	69828681	Gudbjartsson DF et al., 2008	2008	18391951	**European**
727	rs3782415	*SOCS2*	12	93967755	Lanktree MB et al., 2011	2011	21194676	**European**
728	rs3825199	*SOCS2*	12	93976954	Wood AR et al., 2014	2014	25282103	**European**
729	rs11107116	*SOCS2*	12	93978504	He M et al., 2015	2015	25429064	**European**

You created this PDF from an application that is not licensed to print to novaPDF printer (http://www.novapdf.com)

730	rs11107124		12	93988283	Chan Y et al., 2015	2015	25865494	**Various**
731	rs2885691		12	94122219	Yang J et al., 2012	2012	22426310	**European**
732	rs10859567	*CRADD*	12	94126925	Wood AR et al., 2014	2014	25282103	**European**
733	rs7971536	*CCDC53*	12	102373788	Wood AR et al., 2014	2014	25282103	**European**
734	rs2271266	*NUP37*	12	102506044	He M et al., 2015	2015	25429064	**Asian**
735	rs2292303	*NUP37, C12orf48, PMCH*	12	102513531	Kim JJ et al., 2010	2010	19893584	**Korean**
736	rs7313075	*IGF1*	12	102630679	He M et al., 2015	2015	25429064	**Asian**
737	rs12426318	*IGF1*	12	102635521	Kim JJ et al., 2010	2010	19893584	**Korean**
738	rs1520223	*IGF1*	12	102726338	Kim JJ et al., 2010	2010	19893584	**Korean**
739	rs5742692	*IGF1*	12	102799598	Okada Y et al., 2010	2010	20189936	**Japanese**
740	rs2072592	*IGF1*	12	102813632	Kim JJ et al., 2010	2010	19893584	**Korean**
741	rs833706	*PAH*	12	103062597	Wood AR et al., 2014	2014	25282103	**European**
742	rs2164747	*HSP90B1*	12	104344836	Wood AR et al., 2014	2014	25282103	**European**
743	rs2888893	*C12orf23*	12	107338631	Wood AR et al., 2014	2014	25282103	**European**
744	rs11616067	*MED13L*	12	116393174	Wood AR et al., 2014	2014	25282103	**European**
745	rs4767473	*FBXW8*	12	117365506	Wood AR et al., 2014	2014	25282103	**European**
746	rs497273	*SPPL3*	12	121204682	Wood AR et al., 2014	2014	25282103	**European**
747	rs11835818	*BCL7A*	12	122494809	Wood AR et al., 2014	2014	25282103	**European**
748	rs11057552	*FAM101A*	12	124750895	Wood AR et al., 2014	2014	25282103	**European**
749	rs1809889	*FAM101A*	12	124801226	Wood AR et al., 2014	2014	25282103	**European**
750	rs11246833	*LOC729110*	12	132057720	Cho YS et al., 2009	2009	19396169	**Korean**
751	rs1199734	*LATS2*	13	21570246	Wood AR et al., 2014	2014	25282103	**European**
752	rs12323101	*PDS5B*	13	33143406	Wood AR et al., 2014	2014	25282103	**European**
753	rs7332115	*PDS5B/BRCA2*	13	33147548	Lango Allen H et al., 2010	2010	20881960	**European**
754	rs7327412		13	33348568	Yang J et al., 2012	2012	22426310	**European**
755	rs12863103	*STARD13*	13	33723244	Wood AR et al., 2014	2014	25282103	**European**
756	rs6561030	*DGKH*	13	42631719	Okada Y et al., 2010	2010	20189936	**Japanese**

You created this PDF from an application that is not licensed to print to novaPDF printer (http://www.novapdf.com)

757	rs6561319	*LRCH1*	13	47112120	Wood AR et al., 2014	2014	25282103	**European**
758	rs12871822	*CYSLTR2*	13	49201040	Chan Y et al., 2015	2015	25865494	**Various**
759	rs2687950	*KCNRG*	13	50718468	Wood AR et al., 2014	2014	25282103	**European**
760	rs1753637	*DLEU7*	13	51084173	Wood AR et al., 2014	2014	25282103	**European**
761	rs3118905	*DLEU7*	13	51105334	Wood AR et al., 2014	2014	25282103	**European**
762	rs1239947	*DLEU7*	13	51106555	Gudbjartsson DF et al., 2008	2008	18391951	**European**
763	rs3116602	*DLEU7*	13	51111355	Weedon MN et al., 2008	2008	18391952	**European**
764	rs3118912	*DLEU7*	13	51111464	Soranzo N et al., 2009	2009	19343178	**European**
765	rs3118914	*DLEU7*	13	51116901	Soranzo N et al., 2009	2009	19343178	**European**
766	rs3116607	*DLEU7*	13	51122118	Soranzo N et al., 2009	2009	19343178	**European**
767	rs3118916	*DLEU7*	13	51136808	Soranzo N et al., 2009	2009	19343178	**European**
768	rs4883972	*KLF12*	13	75058481	Wood AR et al., 2014	2014	25282103	**European**
769	rs3818416	*EDNRB*	13	78474468	Wood AR et al., 2014	2014	25282103	**European**
770	rs11616380	*SPRY2*	13	80705315	Chan Y et al., 2015	2015	25865494	**Various**
771	rs6563199	*SPRY2*	13	81550449	Chan Y et al., 2015	2015	25865494	**Various**
772	rs8002779	*GPC5*	13	92015977	Okada Y et al., 2010	2010	20189936	**Japanese**
773	rs7319045	*GPC5*	13	92024574	Wood AR et al., 2014	2014	25282103	**European**
774	rs2793701	*RAP2A*	13	98400606	He M et al., 2015	2015	25429064	**East Asian**
775	rs7985356	*CDC16*	13	115027462	Wood AR et al., 2014	2014	25282103	**European**
776	rs8017130	*HOMEZ*	14	23759156	Wood AR et al., 2014	2014	25282103	**European**
777	rs1950500	*NFATC4*	14	24830850	Wood AR et al., 2014	2014	25282103	**European**
778	rs12590407	*NFATC4*	14	24835115	Lanktree MB et al., 2011	2011	21194676	**European**
779	rs12435366	*NFKBIA*	14	35838389	Wood AR et al., 2014	2014	25282103	**European**
780	rs10132817	*NKX2-1, MBIP, NKX2-8, PAX9*	14	36901937	Gudbjartsson DF et al., 2008	2008	18391951	**European**
781	rs17104630	*NKX2-1*	14	37000049	Lettre G et al., 2008	2008	18391950	**European**
782	rs10131337	*PAX9*	14	37144516	Wood AR et al., 2014	2014	25282103	**European**
783	rs6571772	*SLC25A21*	14	37477461	Wood AR et al., 2014	2014	25282103	**European**

You created this PDF from an application that is not licensed to print to novaPDF printer (http://www.novapdf.com)

784	rs8006657	*SAMD4A*	14	55245149	Wood AR et al., 2014	2014	25282103	**European**
785	rs709939	*SAMD4A*	14	55249345	Lanktree MB et al., 2011	2011	21194676	**European**
786	rs11624136	*DAAM1*	14	59688820	Wood AR et al., 2014	2014	25282103	**European**
787	rs2093210	*C14orf39*	14	60957279	Wood AR et al., 2014	2014	25282103	**European**
788	rs2781373	*MAX*	14	65568215	Wood AR et al., 2014	2014	25282103	**European**
789	rs2058092	*NUMB*	14	73932966	Chan Y et al., 2015	2015	25865494	**Various**
790	rs699371	*LTBP2*	14	74989433	He M et al., 2015	2015	25429064	**European**
791	rs862034	*LTBP2*	14	74990746	Wood AR et al., 2014	2014	25282103	**European**
792	rs10140101	*LTBP2*	14	75038689	Chan Y et al., 2015	2015	25865494	**Various**
793	rs910316	*TMED10*	14	75626042	Soranzo N et al., 2009	2009	19343178	**European**
794	rs17110818	*C14orf145*	14	81051353	Cho YS et al., 2009	2009	19396169	**Korean**
795	rs3783937	*FBLN5*	14	92407693	Lettre G et al., 2008	2008	18391950	**European**
796	rs7153027	*FBLN5*	14	92427222	He M et al., 2015	2015	25429064	**European**
797	rs7154721	*TRIP11*	14	92427348	Wood AR et al., 2014	2014	25282103	**European**
798	rs2160077		14	92428410	Yang J et al., 2012	2012	22426310	**European**
799	rs8007661	*TRIP11-ATXN3*	14	92459958	Lettre G et al., 2008	2008	18391950	**European**
800	rs7158300	*TRIP11*	14	92482948	He M et al., 2015	2015	25429064	**European**
801	rs7155279	*TRIP11*	14	92485881	Lango Allen H et al., 2010	2010	20881960	**European**
802	rs1190545	*KIAA0329*	14	102904179	Wood AR et al., 2014	2014	25282103	**European**
803	rs12882130	*MARK3*	14	103878774	Wood AR et al., 2014	2014	25282103	**European**
804	rs10152739	*SPRED1*	15	38483866	Wood AR et al., 2014	2014	25282103	**European**
805	rs1036477	*FBN1*	15	48914926	Wood AR et al., 2014	2014	25282103	**European**
806	rs10744956	*AP4E1*	15	51269629	Wood AR et al., 2014	2014	25282103	**European**
807	rs16964211	*CYP19A1*	15	51530495	Wood AR et al., 2014	2014	25282103	**European**
808	rs2305707	*CYP19A1*	15	51569410	Okada Y et al., 2010	2010	20189936	**Japanese**
809	rs10519302	*CYP19A1*	15	51599683	He M et al., 2015	2015	25429064	**European**
810	rs3751591	*CYP19A1*	15	51606710	Lanktree MB et al., 2011	2011	21194676	**European**
811	rs782930	*RORA*	15	61408362	Wood AR et al., 2014	2014	25282103	**European**
812	rs7177711	*FAM148A*	15	62379971	Wood AR et al., 2014	2014	25282103	**European**

You created this PDF from an application that is not licensed to print to novaPDF printer (http://www.novapdf.com)

813	rs7178424	*C2CD4A*	15	62380259	Lango Allen H et al., 2010	2010	20881960	**European**
814	rs7162825	*LACTB*	15	63439186	Wood AR et al., 2014	2014	25282103	**European**
815	rs17264185	*SMAD6*	15	66997087	Wood AR et al., 2014	2014	25282103	**European**
816	rs975210	*TLE3*	15	70364352	Wood AR et al., 2014	2014	25282103	**European**
817	rs11634405	*THSD4*	15	72084693	Wood AR et al., 2014	2014	25282103	**European**
818	rs12902421	*MYO9A*	15	72161403	Lango Allen H et al., 2010	2010	20881960	**European**
819	rs12904334	*ARIH1*	15	72842705	Wood AR et al., 2014	2014	25282103	**European**
820	rs4337252	*LOXL1*	15	74226765	Wood AR et al., 2014	2014	25282103	**European**
821	rs12440667		15	74231439	Yang J et al., 2012	2012	22426310	**European**
822	rs4886707	*SIN3A-PTPN9*	15	75755467	Okada Y et al., 2010	2010	20189936	**Japanese**
823	rs7184046	*PTPN9*	15	75866150	He M et al., 2015	2015	25429064	**East Asian**
824	rs16968242	*SCAPER*	15	76740219	Wood AR et al., 2014	2014	25282103	**European**
825	rs11858942	*TMED3*	15	79604782	Lettre G et al., 2008	2008	18391950	**European**
826	rs12914466	*TMC3*	15	81836638	Wood AR et al., 2014	2014	25282103	**European**
827	rs2257011	*SH3GL3*	15	84266145	Wood AR et al., 2014	2014	25282103	**European**
828	rs2562784	*SH3GL3-ADAMTSL3*	15	84286492	Lettre G et al., 2008	2008	18391950	**European**
829	rs2554380	*ADAMTSL3, SH3GL3*	15	84315884	Gudbjartsson DF et al., 2008	2008	18391951	**European**
830	rs11853983		15	84462810	Chan Y et al., 2015	2015	25865494	**Various**
831	rs7162542	*ADAMTSL3*	15	84514290	Wood AR et al., 2014	2014	25282103	**European**
832	rs2401171	*ADAMTSL3*	15	84557676	He M et al., 2015	2015	25429064	**European**
833	rs10906982	*ADAMTSL3*	15	84568158	Weedon MN et al., 2008	2008	18391952	**European**
834	rs7183263	*ADAMTSL3*	15	84573041	Okada Y et al., 2010	2010	20189936	**Japanese**
835	rs11259936	*ADAMTSL3*	15	84580582	Lango Allen H et al., 2010	2010	20881960	**European**
836	rs4842838	*ADAMTSL3*	15	84582124	Soranzo N et al., 2009	2009	19343178	**European**
837	rs2011013		15	84625766	Chan Y et al., 2015	2015	25865494	**Various**
838	rs12148239		15	84641018	Yang J et al., 2012	2012	22426310	**European**
839	rs11855014	*PDE8A*	15	85728834	Wood AR et al., 2014	2014	25282103	**European**

You created this PDF from an application that is not licensed to print to novaPDF printer (http://www.novapdf.com)

840	rs1348002	*DET1*	15	89113138	Wood AR et al., 2014	2014	25282103	**European**
841	rs11633371	*ACAN*	15	89356832	Wood AR et al., 2014	2014	25282103	**European**
842	rs8041863	*ACAN*	15	89359689	Weedon MN et al., 2008	2008	18391952	**European**
843	rs4932429		15	89363532	Yang J et al., 2012	2012	22426310	**European**
844	rs2280470	*ACAN*	15	89395626	Wood AR et al., 2014	2014	25282103	**European**
845	rs2238300	*FANCI*	15	89851580	Wood AR et al., 2014	2014	25282103	**European**
846	rs8028843	*RGMA*	15	94028149	Chan Y et al., 2015	2015	25865494	**Various**
847	rs7181724	*MCTP2*	15	94551607	Wood AR et al., 2014	2014	25282103	**European**
848	rs2871865	*IGF1R*	15	99194896	He M et al., 2015	2015	25429064	**European**
849	rs2573625	*ADAMTS17*	15	100513158	Wood AR et al., 2014	2014	25282103	**European**
850	rs2573652	*ADAMTS17*	15	100514614	He M et al., 2015	2015	25429064	**European**
851	rs12916269		15	100530216	Yang J et al., 2012	2012	22426310	**European**
852	rs4246302	*ADAMTS17*	15	100687967	Wood AR et al., 2014	2014	25282103	**European**
853	rs2035344		15	100690148	Yang J et al., 2012	2012	22426310	**European**
854	rs4965598	*ADAMTS17*	15	100759614	Lango Allen H et al., 2010	2010	20881960	**European**
855	rs4548838	*ADAMTS17*	15	100761190	Wood AR et al., 2014	2014	25282103	**European**
856	rs4533267	*ADAMTS17*	15	100786271	Gudbjartsson DF et al., 2008	2008	18391951	**European**
857	rs7170986	*LRRK1*	15	101632867	Wood AR et al., 2014	2014	25282103	**European**
858	rs8042424	*CHSY1*	15	101762539	Wood AR et al., 2014	2014	25282103	**European**
859	rs763014	*RAB40C*	16	675680	Lettre G et al., 2008	2008	18391950	**European**
860	rs12597498	*LMF1*	16	990815	Wood AR et al., 2014	2014	25282103	**European**
861	rs2014467	*ABCA3*	16	2336394	Wood AR et al., 2014	2014	25282103	**European**
862	rs129963	*CREBBP*	16	3796147	Wood AR et al., 2014	2014	25282103	**European**
863	rs960006	*UBN1*	16	4911195	Wood AR et al., 2014	2014	25282103	**European**
864	rs1659127	*MKL2-PARN*	16	14388305	He M et al., 2015	2015	25429064	**European**
865	rs1136001	*PDXDC1-NTAN1*	16	15131974	Okada Y et al., 2010	2010	20189936	**Japanese**
866	rs2023693	*DCUN1D3*	16	20880040	Wood AR et al., 2014	2014	25282103	**European**

You created this PDF from an application that is not licensed to print to novaPDF printer (http://www.novapdf.com)

867	rs11642612	*FLJ25404*	16	30030195	Wood AR et al., 2014	2014	25282103	**European**
868	rs4785393	*PAPD5*	16	50259483	Wood AR et al., 2014	2014	25282103	**European**
869	rs9929889	*SALL1*	16	51094038	Wood AR et al., 2014	2014	25282103	**European**
870	rs8058684	*RBL2*	16	53515118	Wood AR et al., 2014	2014	25282103	**European**
871	rs604129		16	66704310	Chan Y et al., 2015	2015	25865494	**Various**
872	rs3790086	*WWP2*	16	69887707	Wood AR et al., 2014	2014	25282103	**European**
873	rs217181	*HPR*	16	72114002	Wood AR et al., 2014	2014	25282103	**European**
874	rs11640018	*CFDP1*	16	75328308	Wood AR et al., 2014	2014	25282103	**European**
875	rs7189843	*PLCG2*	16	81902139	Cho YS et al., 2009	2009	19396169	**Korean**
876	rs6420435	*MPHOSPH6*	16	82184201	Wood AR et al., 2014	2014	25282103	**European**
877	rs6563943	*CDH13*	16	83639335	Okada Y et al., 2010	2010	20189936	**Japanese**
878	rs2326458	*ZDHHC7*	16	84987679	Wood AR et al., 2014	2014	25282103	**European**
879	rs4843367	*FOXF1*	16	86417890	Wood AR et al., 2014	2014	25282103	**European**
880	rs300039	*FOXL1*	16	86688976	Chan Y et al., 2015	2015	25865494	**Various**
881	rs2377058	*C16orf55*	16	89734831	Chan Y et al., 2015	2015	25865494	**Various**
882	rs258324	*CDK10*	16	89754255	He M et al., 2015	2015	25429064	**East Asian**
883	rs870183	*VPS53*	17	599811	Wood AR et al., 2014	2014	25282103	**European**
884	rs9217	*ZBTB4*	17	7363088	Wood AR et al., 2014	2014	25282103	**European**
885	rs8071847	*POLR2A*	17	7407327	Lanktree MB et al., 2011	2011	21194676	**European**
886	rs8073177	*TNFSF12*	17	7440584	Chan Y et al., 2015	2015	25865494	**Various**
887	rs2270518	*KDM6B*	17	7758522	He M et al., 2015	2015	25429064	**East Asian**
888	rs8069300	*MAP2K4*	17	11984232	Wood AR et al., 2014	2014	25282103	**European**
889	rs3110496	*ANKRD13B*	17	27917771	Lango Allen H et al., 2010	2010	20881960	**European**
890	rs871014		17	27945339	Yang J et al., 2012	2012	22426310	**European**
891	rs3809790	*SSH2*	17	27955540	Chan Y et al., 2015	2015	25865494	**Various**
892	rs3764419	*ATAD5/RNF135*	17	29164023	Lango Allen H et al., 2010	2010	20881960	**European**
893	rs9889755	*C17orf42*	17	29234505	Chan Y et al., 2015	2015	25865494	**Various**
894	rs3760318	*CENTA2*	17	29247715	Wood AR et al., 2014	2014	25282103	**European**
895	rs2028067	*UTP6*	17	30239698	Wood AR et al., 2014	2014	25282103	**European**

You created this PDF from an application that is not licensed to print to novaPDF printer (http://www.novapdf.com)

896	rs1043515	*PIP4K2B*	17	36922196	Lango Allen H et al., 2010	2010	20881960	**European**
897	rs2338115	*PIP4K2B*	17	36929578	Wood AR et al., 2014	2014	25282103	**European**
898	rs584828	*IGFBP4*	17	38599230	Wood AR et al., 2014	2014	25282103	**European**
899	rs2315504	*KRT23, KRT20*	17	39046881	He M et al., 2015	2015	25429064	**East Asian**
900	rs16966703	*KRT33A*	17	39502398	Lei SF et al., 2009	2009	19039035	**Chinese**
901	rs9766	*EZH1*	17	40852841	Wood AR et al., 2014	2014	25282103	**European**
902	rs4986172	*ACBD4*	17	43216281	Wood AR et al., 2014	2014	25282103	**European**
903	rs6504389	*HOXB3*	17	46643364	Chan Y et al., 2015	2015	25865494	**Various**
904	rs318095	*ATP5G1*	17	46974734	Wood AR et al., 2014	2014	25282103	**European**
905	rs8182364		17	47018025	Yang J et al., 2012	2012	22426310	**European**
906	rs2072153	*ZNF652*	17	47390014	Wood AR et al., 2014	2014	25282103	**European**
907	rs4605213	*NME1-NME2/NME2*	17	49244747	Wood AR et al., 2014	2014	25282103	**European**
908	rs11867943	*ANKFN1*	17	54229842	Wood AR et al., 2014	2014	25282103	**European**
909	rs12449568	*ANKFN1*	17	54430155	Lettre G et al., 2008	2008	18391950	**European**
910	rs227724	*C17orf67*	17	54778817	Wood AR et al., 2014	2014	25282103	**European**
911	rs1401795	*C17orf67*	17	54839652	Wood AR et al., 2014	2014	25282103	**European**
912	rs4794665		17	54850329	Yang J et al., 2012	2012	22426310	**European**
913	rs9892365	*TBX2*	17	59491384	Lanktree MB et al., 2011	2011	21194676	**European**
914	rs758598	*C17orf82, TBX4*	17	59492714	Kim JJ et al., 2010	2010	19893584	**Korean**
915	rs1076392	*C17orf82, TBX4*	17	59493008	Kim JJ et al., 2010	2010	19893584	**Korean**
916	rs882367		17	59494574	Chan Y et al., 2015	2015	25865494	**Various**
917	rs2079795	*C17orf82*	17	59496649	Wood AR et al., 2014	2014	25282103	**European**
918	rs757608	*TBX4*	17	59497277	He M et al., 2015	2015	25429064	**European**
919	rs2378870	*NACA2*	17	59638623	Wood AR et al., 2014	2014	25282103	**European**
920	rs12451513		17	59642328	Yang J et al., 2012	2012	22426310	**European**
921	rs7209435	*MAP3K3, WDR68, LYK5, MT1F*	17	61712964	Gudbjartsson DF et al., 2008	2008	18391951	**European**

You created this PDF from an application that is not licensed to print to novaPDF printer (http://www.novapdf.com)

922	rs8081612	*MAP3K3*	17	61724695	Lanktree MB et al., 2011	2011	21194676	**European**
923	rs12325866	*MAP3K3*	17	61755974	Soranzo N et al., 2009	2009	19343178	**European**
924	rs3785574	*MAP3K3*	17	61763185	He M et al., 2015	2015	25429064	**European, East Asian**
925	rs2854207	*CSH2*	17	61947107	Wood AR et al., 2014	2014	25282103	**European**
926	rs2854160	*CSH1*	17	61977248	He M et al., 2015	2015	25429064	**European**
927	rs7921	*GH1-GH2*	17	62006259	Lanktree MB et al., 2011	2011	21194676	**European**
928	rs2070776	*CD79B*	17	62007498	Wood AR et al., 2014	2014	25282103	**European**
929	rs3923086	*AXIN2*	17	63549488	Wood AR et al., 2014	2014	25282103	**European**
930	rs2072268	*ARSG*	17	66303352	Wood AR et al., 2014	2014	25282103	**European**
931	rs11867479	*KCNJ16*	17	68090207	Wood AR et al., 2014	2014	25282103	**European**
932	rs10083886	*SOX9*	17	69923355	Wood AR et al., 2014	2014	25282103	**European**
933	rs2158917		17	69926109	Yang J et al., 2012	2012	22426310	**European**
934	rs2117563	*GRB2*	17	73368985	Wood AR et al., 2014	2014	25282103	**European**
935	rs959260	*GRB2*	17	73369422	Lanktree MB et al., 2011	2011	21194676	**European**
936	rs1552173	*PSCD1*	17	76718842	Wood AR et al., 2014	2014	25282103	**European**
937	rs2279308		17	76794981	Yang J et al., 2012	2012	22426310	**European**
938	rs4239020	*CCDC57*	17	80176641	Wood AR et al., 2014	2014	25282103	**European**
939	rs692964	*CEP192*	18	13094132	Wood AR et al., 2014	2014	25282103	**European**
940	rs4800367		18	18637097	Chan Y et al., 2015	2015	25865494	**Various**
941	rs291794		18	18983118	Chan Y et al., 2015	2015	25865494	**Various**
942	rs2850575		18	19235006	Chan Y et al., 2015	2015	25865494	**Various**
943	rs14062	*MIB1*	18	19450303	Wood AR et al., 2014	2014	25282103	**European**
944	rs8098316	*RBBP8*	18	20672555	He M et al., 2015	2015	25429064	**European, East Asian**
945	rs4800148	*CABLES1, RBBP8, C18orf45*	18	20724328	Gudbjartsson DF et al., 2008	2008	18391951	**European**
946	rs4800452	*CABLES1*	18	20727611	Lango Allen H et al., 2010	2010	20881960	**European**
947	rs4369779	*CABLES1*	18	20735408	He M et al., 2015	2015	25429064	**European**
948	rs4308051		18	20735461	Chan Y et al., 2015	2015	25865494	**Various**

You created this PDF from an application that is not licensed to print to novaPDF printer (http://www.novapdf.com)

949	rs8094261		18	20746728	Kim JJ et al., 2010	2010	19893584	**Korean**
950	rs16958440		18	44632884	Chan Y et al., 2015	2015	25865494	**Various**
951	rs11661645	*KIAA0427*	18	45888770	Wood AR et al., 2014	2014	25282103	**European**
952	rs12454567	*KIAA0427*	18	46270114	Wood AR et al., 2014	2014	25282103	**European**
953	rs2337143	*SMAD7*	18	46482070	Wood AR et al., 2014	2014	25282103	**European**
954	rs16950303	*DYM*	18	46582359	He M et al., 2015	2015	25429064	**European, East Asian**
955	rs12458127	*DYM*	18	46657358	Wood AR et al., 2014	2014	25282103	**European**
956	rs8099594	*DYM*	18	46991160	Weedon MN et al., 2008	2008	18391952	**European**
957	rs12958987	*DCC*	18	50359002	Lettre G et al., 2008	2008	18391950	**European**
958	rs8098032		18	53239302	Chan Y et al., 2015	2015	25865494	**Various**
959	rs12458596		18	53410912	Chan Y et al., 2015	2015	25865494	**Various**
960	rs17782313	*MC4R*	18	57851097	Lango Allen H et al., 2010	2010	20881960	**European**
961	rs10871777		18	57851763	Yang J et al., 2012	2012	22426310	**European**
962	rs11152213	*MC4R*	18	57852948	Wood AR et al., 2014	2014	25282103	**European**
963	rs8097893	*GALR1*	18	74983055	Wood AR et al., 2014	2014	25282103	**European**
964	rs11659752	*NFATC1*	18	77222862	Wood AR et al., 2014	2014	25282103	**European**
965	rs12986413	*DOT1L*	19	2170954	He M et al., 2015	2015	25429064	**East Asian**
966	rs11880992	*DOT1L*	19	2176403	Wood AR et al., 2014	2014	25282103	**European**
967	rs12982744	*DOT1L*	19	2177193	Lango Allen H et al., 2010	2010	20881960	**European**
968	rs2123731	*UHRF1*	19	4929473	Wood AR et al., 2014	2014	25282103	**European**
969	rs8108622	*INSR*	19	7182753	Lanktree MB et al., 2011	2011	21194676	**European**
970	rs891088	*INSR*	19	7184762	Wood AR et al., 2014	2014	25282103	**European**
971	rs10413734		19	7227871	Yang J et al., 2012	2012	22426310	**European**
972	rs1346490	*INSR*	19	7244233	Wood AR et al., 2014	2014	25282103	**European**
973	rs7249094	*ADAMTS10, MYO1F, PRAM1, OR2Z1*	19	8672000	Gudbjartsson DF et al., 2008	2008	18391951	**European**
974	rs2228612	*ADAMTS10*	19	10273372	Lanktree MB et al., 2011	2011	21194676	**European**
975	rs6511689	*S1PR2*	19	10321089	Wood AR et al., 2014	2014	25282103	**European**

You created this PDF from an application that is not licensed to print to novaPDF printer (http://www.novapdf.com)

976	rs7250071	*ILF3*	19	10765819	He M et al., 2015	2015	25429064	**East Asian**
977	rs8102380	*ILF3*	19	10801185	Wood AR et al., 2014	2014	25282103	**European**
978	rs12459943	*DNM2*	19	10859508	He M et al., 2015	2015	25429064	**East Asian**
979	rs7259684	*LOC729747*	19	12186611	Chan Y et al., 2015	2015	25865494	**Various**
980	rs2279008	*MYO9B*	19	17283303	Lango Allen H et al., 2010	2010	20881960	**European**
981	rs10401193	*GATAD2A*	19	19591066	Wood AR et al., 2014	2014	25282103	**European**
982	rs4802134	*SIPA1L3*	19	38346685	Wood AR et al., 2014	2014	25282103	**European**
983	rs4803468	*BCKDHA*	19	41922352	Wood AR et al., 2014	2014	25282103	**European**
984	rs17318596	*ATP5SL*	19	41937095	Lango Allen H et al., 2010	2010	20881960	**European**
985	rs11880124	*DEDD2*	19	42683791	Wood AR et al., 2014	2014	25282103	**European**
986	rs7273787	*SMOX*	20	4098567	Wood AR et al., 2014	2014	25282103	**European**
987	rs1741344	*SMOX*	20	4101800	Lango Allen H et al., 2010	2010	20881960	**European**
988	rs17721822	*BMP2*	20	6469596	Wood AR et al., 2014	2014	25282103	**European**
989	rs1884897	*BMP2*	20	6612832	Wood AR et al., 2014	2014	25282103	**European**
990	rs967417	*BMP2*	20	6620893	Gudbjartsson DF et al., 2008	2008	18391951	**European**
991	rs2145272	*BMP2*	20	6626218	He M et al., 2015	2015	25429064	**European**
992	rs6140050		20	6632901	Yang J et al., 2012	2012	22426310	**European**
993	rs6085662	*BMP2*	20	6698372	Wood AR et al., 2014	2014	25282103	**European**
994	rs6080830	*BANF2*	20	17771113	Wood AR et al., 2014	2014	25282103	**European**
995	rs7261425	*C20orf26*	20	20068635	Wood AR et al., 2014	2014	25282103	**European**
996	rs8117259	*INSM1*	20	20348253	Wood AR et al., 2014	2014	25282103	**European**
997	rs6137287	*C20orf19*	20	21180259	Wood AR et al., 2014	2014	25282103	**European**
998	rs291700	*CDK5RAP1*	20	31981849	Lanktree MB et al., 2011	2011	21194676	**European**
999	rs1074683	*PXMP4*	20	32304653	Wood AR et al., 2014	2014	25282103	**European**
1000	rs7274811	*ZNF341*	20	32333181	Lango Allen H et al., 2010	2010	20881960	**European**
1001	rs2425012	*MYH7B*	20	33581955	Lanktree MB et al., 2011	2011	21194676	**European**
1002	rs6060154		20	33599601	Yang J et al., 2012	2012	22426310	**European**
1003	rs1535466	*EDEM2*	20	33718706	Wood AR et al., 2014	2014	25282103	**European**

You created this PDF from an application that is not licensed to print to novaPDF printer (http://www.novapdf.com)

1004	rs2425019	*MMP24*	20	33819415	Lanktree MB et al., 2011	2011	21194676	**European**
1005	rs6060369	*UQCC*	20	33907161	He M et al., 2015	2015	25429064	**European**
1006	rs6088792	*UQCC, GDF5, CEP250, EIF6, MMP24*	20	33909784	Gudbjartsson DF et al., 2008	2008	18391951	**European**
1007	rs6060373	*GDF5*	20	33914208	Weedon MN et al., 2008	2008	18391952	**European**
1008	rs4911494	*UQCC*	20	33971914	Soranzo N et al., 2009	2009	19343178	**European**
1009	rs6088813	*UQCC*	20	33975181	Soranzo N et al., 2009	2009	19343178	**European**
1010	rs224329	*GDF5*	20	34019579	He M et al., 2015	2015	25429064	**European**
1011	rs143384	*GDF5*	20	34025756	Wood AR et al., 2014	2014	25282103	**European**
1012	rs2236164	*CEP250*	20	34097353	He M et al., 2015	2015	25429064	**European**
1013	rs2425163	*PHF20*	20	34432670	Wood AR et al., 2014	2014	25282103	**European**
1014	rs6060739		20	34567592	Yang J et al., 2012	2012	22426310	**European**
1015	rs4812586	*SAMHD1*	20	35544673	Wood AR et al., 2014	2014	25282103	**European**
1016	rs6030712	*RBL1*	20	35637398	He M et al., 2015	2015	25429064	**East Asian**
1017	rs2224538	*MAFB*	20	38552078	Chan Y et al., 2015	2015	25865494	**Various**
1018	rs17450430	*STAU1*	20	47772264	Wood AR et al., 2014	2014	25282103	**European**
1019	rs6020202	*SNAI1*	20	48634821	Wood AR et al., 2014	2014	25282103	**European**
1020	rs913000		20	54836354	Yang J et al., 2012	2012	22426310	**European**
1021	rs1326023	*MC3R*	20	54842378	Wood AR et al., 2014	2014	25282103	**European**
1022	rs4811971	*ANKRD60*	20	56796784	He M et al., 2015	2015	25429064	**East Asian**
1023	rs2057291	*GNAS*	20	57472043	Wood AR et al., 2014	2014	25282103	**European**
1024	rs6061231	*RPS21*	20	60956917	Wood AR et al., 2014	2014	25282103	**European**
1025	rs2829941	*APP*	21	27208935	Wood AR et al., 2014	2014	25282103	**European**
1026	rs2834442	*KCNE2*	21	35690786	Wood AR et al., 2014	2014	25282103	**European**
1027	rs2211866	*KCNJ15*	21	39688107	Wood AR et al., 2014	2014	25282103	**European**
1028	rs9980072		21	40338817	Chan Y et al., 2015	2015	25865494	**Various**
1029	rs9977276	*COL6A1*	21	47436327	Wood AR et al., 2014	2014	25282103	**European**

You created this PDF from an application that is not licensed to print to novaPDF printer (http://www.novapdf.com)

1030	rs5751614	*BCR, GNAZ, RTDR1, IGLL1*	22	23593051	Gudbjartsson DF et al., 2008	2008	18391951	**European**
1031	rs7284476	*TRIOBP*	22	38129332	Wood AR et al., 2014	2014	25282103	**European**
1032	rs5757318	*CBX6*	22	39275656	Wood AR et al., 2014	2014	25282103	**European**
1033	rs11090631	*RIBC2*	22	45846371	Wood AR et al., 2014	2014	25282103	**European**

You created this PDF from an application that is not licensed to print to novaPDF printer (http://www.novapdf.com)

Table S2 | 34 human height GWAS genetic SNPs were associated with FSS (*p* < 5.00E-05 (0.05/1,033) under the additive model)

	rs ID	Gene	Chr.	Chr. position	Risk allele	Additive model		Dominance deviation from additivity (DOMDEV)		Reference	Year	PMID number	Population
						OR (95% CI)	*P*	OR (95% CI)	*P*				
1	rs1926872	*COLGALT2 / TSEN15*	1	184,049,341	T	1.33 (1.19-1.52)	2.89E-06	1.16 (0.98-1.38)	9.40E-02	He M et al., 2015	2015	25429064	European
2	rs1046934	*TSEN15*	1	184,054,395	A	1.32 (1.16-1.49)	1.06E-05	1.14 (0.96-1.35)	1.50E-01	Lango Allen H et al., 2010	2010	20881960	European
3	rs3791679	*EFEMP1*	2	55,869,757	C	1.39 (1.19-1.61)	1.96E-05	1.09 (0.83-1.41)	5.43E-01	Wood AR et al., 2014	2014	25282103	European
4	rs3791675	*EFEMP1*	2	55,884,174	T	1.37 (1.19-1.59)	2.81E-05	1.01 (0.78-1.32)	9.13E-01	He M et al., 2015	2015	25429064	European, Asian
5	rs3771381	*ZNF638*	2	71,333,535	A	1.31 (1.16-1.48)	1.83E-05	1.08 (0.91-1.29)	3.93E-01	He M et al., 2015	2015	25429064	East Asian
6	rs10935120	*CEP63*	3	134,514,250	A	1.43 (1.22-1.68)	1.27E-05	1.05 (0.78-1.42)	7.37E-01	Weedon MN et al., 2008	2008	18391952	European
7	rs6440003	*ZBTB38*	3	141,375,367	G	1.33 (1.16-1.52)	2.60E-05	1.01 (0.82-1.23)	9.63E-01	Weedon MN et al., 2008	2008	18391952	European
8	rs7632381	*ZBTB38*	3	141,387,221	T	1.35 (1.18-1.54)	1.17E-05	1.04 (0.85-1.27)	7.37E-01	Kim JJ et al., 2010	2010	19893584	Korean
9	rs1344672	*ZBTB38*	3	141,406,863	C	1.32 (1.15-1.52)	4.11E-05	1.02 (0.84-1.25)	8.18E-01	Kim JJ et al., 2010	2010	19893584	Korean
10	rs9825379	*ZBTB38*	3	141,418,193	G	1.45 (1.22-1.69)	1.24E-05	1.04 (0.76-1.43)	8.03E-01	Okada Y et al., 2010	2010	20189936	Japanese
11	rs7678436	*DCAF16*	4	17,796,343	A	1.40 (1.24-1.60)	1.77E-07	1.04 (0.86-1.25)	7.03E-01	Okada Y et al., 2010	2010	20189936	Japanese
12	rs16895802	*NCAPG*	4	17,814,266	G	1.48 (1.24-1.76)	1.36E-05	1.17 (0.82-1.67)	3.81E-01	He M et al., 2015	2015	25429064	European, Asian
13	rs6842303	*LCORL*	4	17,852,432	G	1.28 (1.14-1.45)	4.25E-05	1.10 (0.92-1.30)	2.98E-01	Gudbjartsson DF et al., 2008	2008	18391951	European
14	rs13131350	*LCORL*	4	17,875,864	G	1.55 (1.36-1.78)	2.06E-10	1.05 (0.85-1.30)	6.59E-01	He M et al., 2015	2015	25429064	European, Asian
15	rs16896276	*LCORL*	4	18,013,533	T	1.32 (1.16-1.49)	1.13E-05	1.12 (0.94-1.33)	1.90E-01	Yang J et al., 2012	2012	22426310	European
16	rs2011603	*LCORL*	4	18,023,861	A	1.30 (1.15-1.47)	3.23E-05	1.15 (0.97-1.37)	1.14E-01	Cho YS et al., 2009	2009	19396169	Korean
17	rs17720281	*NA*	4	144,622,624	C	1.41 (1.22-1.64)	6.51E-06	1.07 (0.82-1.39)	6.11E-01	Yang J et al., 2012	2012	22426310	European
18	rs6845999	*HHIP*	4	144,644,674	C	1.41 (1.22-1.64)	6.86E-06	1.03 (0.79-1.34)	8.12E-01	Wood AR et al., 2014	2014	25282103	European
19	rs4240326	*ANAPC10*	4	144,918,112	G	1.37 (1.19-1.59)	5.95E-06	1.01 (0.81-1.26)	9.18E-01	Wood AR et al., 2014	2014	25282103	European
20	rs6823268	*ANAPC10*	4	145,061,411	A	1.49 (1.25-1.79)	7.85E-06	1.09 (0.74-1.61)	6.56E-01	He M et al., 2015	2015	25429064	European
21	rs4733724	*GSDMC*	8	129,711,482	G	1.33 (1.16-1.54)	3.35E-05	1.08 (0.86-1.35)	5.10E-01	Wood AR et al., 2014	2014	25282103	European
22	rs6470764	*GSDMC*	8	129,713,419	T	1.35 (1.18-1.54)	2.57E-05	1.09 (0.87-1.37)	4.59E-01	Lango Allen H et al., 2010	2010	20881960	European
23	rs10858250	*QSOX2*	9	136,227,369	A	1.37 (1.18-1.59)	4.62E-05	1.30 (1.01-1.67)	4.49E-02	He M et al., 2015	2015	25429064	European, Asian

You created this PDF from an application that is not licensed to print to novaPDF printer (http://www.novapdf.com)

24	rs12338076	*QSOX2*	9	136,229,894	A	1.33 (1.16-1.52)	2.92E-05	1.25 (1.01-1.54)	3.93E-02	Okada Y et al., 2010	2010	20189936	Japanese
25	rs2401171	*ADAMTSL3*	15	83,888,924	T	1.41 (1.23-1.62)	1.18E-06	1.23 (0.98-1.54)	7.14E-02	He M et al., 2015	2015	25429064	European
26	rs10906982	*ADAMTSL3*	15	83,899,406	T	1.39 (1.21-1.60)	3.24E-06	1.23 (0.98-1.54)	7.31E-02	Weedon MN et al., 2008	2008	18391952	European
27	rs7183263	*ADAMTSL3*	15	83,904,289	T	1.41 (1.23-1.62)	1.00E-06	1.22 (0.98-1.54)	7.58E-02	Okada Y et al., 2010	2010	20189936	Japanese
28	rs11259936	*ADAMTSL3*	15	83,911,830	A	1.41 (1.23-1.62)	1.21E-06	1.23 (0.98-1.54)	7.09E-02	Lango Allen H et al., 2010	2010	20881960	European
29	rs4842838	*ADAMTSL3*	15	83,913,372	G	1.42 (1.23-1.63)	8.51E-07	1.23 (0.99-1.56)	6.25E-02	Soranzo N et al., 2009	2009	19343178	European
30	rs258324	*CDK10*	16	89,687,847	C	1.37 (1.19-1.56)	7.32E-06	1.02 (0.83-1.27)	8.27E-01	He M et al., 2015	2015	25429064	East Asian
31	rs4800452	*CABLES1*	18	23,147,647	C	1.39 (1.19-1.62)	3.32E-05	1.08 (0.81-1.43)	6.16E-01	Lango Allen H et al., 2010	2010	20881960	European
32	rs4369779	*CABLES1*	18	23,155,444	T	1.43 (1.21-1.68)	1.76E-05	1.05 (0.78-1.42)	7.45E-01	He M et al., 2015	2015	25429064	European
33	rs4308051	*CABLES1*	18	23,155,497	T	1.43 (1.22-1.69)	1.39E-05	1.06 (0.78-1.43)	7.21E-01	Chan Y et al., 2015	2015	25865494	Various
34	rs8094261	*CABLES1*	18	23,166,764	G	1.43 (1.22-1.67)	1.40E-05	1.05 (0.78-1.41)	7.58E-01	Kim JJ et al., 2010	2010	19893584	Korean

Abbreviations: SNP, single nucleotide polymorphism; GWAS, genome-wide association studies; chr., chromosome; OR, odds ratio; CI, confidence interval.

Significant p value for the DOMDEV test < 1.47E-3 (0.05/34).

You created this PDF from an application that is not licensed to print to novaPDF printer (http://www.novapdf.com)

Table S3 | Linkage disequilibrium coefficients (D' and r^2) of the single nucleotide polymorphisms located in chromosome 1, 2 and 3

	No.	rs ID	Gene	Chr.	Position	Chr. 1				Chr. 2						Chr. 3									
						rs1926872		rs1046934		rs3791679		rs3791675		rs3771381		rs10935120		rs6440003		rs7632381		rs1344672		rs9825379	
						D'	R^2	D'	R^2	D'	R^2	D'	R^2	D'	R^2	D'	R^2	D'	R^2	D'	R^2	D'	R^2	D'	R^2
FSS	1	rs1926872	*COLGALT2 / TSEN15*	1	184049341	ND	ND	0.994	0.961	Unrelated	Unrelated	Unrelated	Unrelated	Unrelated	Unrelated	Unrelated	Unrelated	Unrelated	Unrelated	Unrelated	Unrelated	Unrelated	Unrelated	Unrelated	Unrelated
	2	rs1046934	*TSEN15*	1	184054395	0.994	0.961	ND	ND	Unrelated	Unrelated	Unrelated	Unrelated	Unrelated	Unrelated	Unrelated	Unrelated	Unrelated	Unrelated	Unrelated	Unrelated	Unrelated	Unrelated	Unrelated	Unrelated
	3	rs3791679	*EFEMP1*	2	55869757	Unrelated	Unrelated	Unrelated	Unrelated	ND	ND	0.983	0.942	Unrelated	Unrelated	Unrelated	Unrelated	Unrelated	Unrelated	Unrelated	Unrelated	Unrelated	Unrelated	Unrelated	Unrelated
	4	rs3791675	*EFEMP1*	2	55884174	Unrelated	Unrelated	Unrelated	Unrelated	0.983	0.942	ND	ND	Unrelated	Unrelated	Unrelated	Unrelated	Unrelated	Unrelated	Unrelated	Unrelated	Unrelated	Unrelated	Unrelated	Unrelated
	5	rs3771381	*ZNF638*	2	71333535	Unrelated	Unrelated	Unrelated	Unrelated	Unrelated	Unrelated	Unrelated	Unrelated	ND	ND	Unrelated	Unrelated	Unrelated	Unrelated	Unrelated	Unrelated	Unrelated	Unrelated	Unrelated	Unrelated
	6	rs10935120	*CEP63*	3	134514250	Unrelated	Unrelated	Unrelated	Unrelated	Unrelated	Unrelated	Unrelated	Unrelated	Unrelated	Unrelated	Unrelated	Unrelated	Unrelated	Unrelated	Unrelated	Unrelated	Unrelated	Unrelated	Unrelated	Unrelated
	7	rs6440003	*ZBTB38*	3	141375367	Unrelated	Unrelated	Unrelated	Unrelated	Unrelated	Unrelated	Unrelated	Unrelated	Unrelated	Unrelated	Unrelated	Unrelated	ND	ND	0.990	0.953	0.990	0.942	0.936	0.376
	8	rs7632381	*ZBTB38*	3	141387221	Unrelated	Unrelated	Unrelated	Unrelated	Unrelated	Unrelated	Unrelated	Unrelated	Unrelated	Unrelated	Unrelated	Unrelated	0.990	0.953	ND	ND	1.000	0.988	0.953	0.379
	9	rs1344672	*ZBTB38*	3	141406863	Unrelated	Unrelated	Unrelated	Unrelated	Unrelated	Unrelated	Unrelated	Unrelated	Unrelated	Unrelated	Unrelated	Unrelated	0.990	0.942	1.000	0.988	ND	ND	0.959	0.379
	10	rs9825379	*ZBTB38*	3	141418193	Unrelated	Unrelated	Unrelated	Unrelated	Unrelated	Unrelated	Unrelated	Unrelated	Unrelated	Unrelated	Unrelated	Unrelated	0.936	0.376	0.953	0.379	0.959	0.379	ND	ND
Control	1	rs1926872	*COLGALT2*	1	184049341	ND	ND	0.996	0.979	Unrelated	Unrelated	Unrelated	Unrelated	Unrelated	Unrelated	Unrelated	Unrelated	Unrelated	Unrelated	Unrelated	Unrelated	Unrelated	Unrelated	Unrelated	Unrelated

You created this PDF from an application that is not licensed to print to novaPDF printer (http://www.novapdf.com)

2	rs1046934	*TSEN15*	1	184054395	0.996	0.979	ND	ND	Unrelated	Unrelated	Unrelated	Unrelated	Unrelated	Unrelated	Unrelated	Unrelated	Unrelated	Unrelated	Unrelated	Unrelated	Unrelated	Unrelated	Unrelated	Unrelated
3	rs3791679	*EFEMP1*	2	55869757	Unrelated	Unrelated	Unrelated	Unrelated	ND	ND	0.976	0.937	Unrelated	Unrelated	Unrelated	Unrelated	Unrelated	Unrelated	Unrelated	Unrelated	Unrelated	Unrelated	Unrelated	Unrelated
4	rs3791675	*EFEMP1*	2	55884174	Unrelated	Unrelated	Unrelated	Unrelated	0.976	0.937	ND	ND	Unrelated	Unrelated	Unrelated	Unrelated	Unrelated	Unrelated	Unrelated	Unrelated	Unrelated	Unrelated	Unrelated	Unrelated
5	rs3771381	*ZNF638*	2	71333535	Unrelated	Unrelated	Unrelated	Unrelated	Unrelated	Unrelated	Unrelated	Unrelated	ND	ND	Unrelated	Unrelated	Unrelated	Unrelated	Unrelated	Unrelated	Unrelated	Unrelated	Unrelated	Unrelated
6	rs10935120	*CEP63*	3	134514250	Unrelated	Unrelated	Unrelated	Unrelated	Unrelated	Unrelated	Unrelated	Unrelated	Unrelated	Unrelated	Unrelated	Unrelated	Unrelated	Unrelated	Unrelated	Unrelated	Unrelated	Unrelated	Unrelated	Unrelated
7	rs6440003	*ZBTB38*	3	141375367	Unrelated	Unrelated	Unrelated	Unrelated	Unrelated	Unrelated	Unrelated	Unrelated	Unrelated	Unrelated	Unrelated	Unrelated	ND	ND	0.990	0.945	0.984	0.939	0.959	0.428
8	rs7632381	*ZBTB38*	3	141387221	Unrelated	Unrelated	Unrelated	Unrelated	Unrelated	Unrelated	Unrelated	Unrelated	Unrelated	Unrelated	Unrelated	Unrelated	0.990	0.945	ND	ND	1.000	0.994	0.971	0.423
9	rs1344672	*ZBTB38*	3	141406863	Unrelated	Unrelated	Unrelated	Unrelated	Unrelated	Unrelated	Unrelated	Unrelated	Unrelated	Unrelated	Unrelated	Unrelated	0.984	0.939	1.000	0.994	ND	ND	0.971	0.425
10	rs9825379	*ZBTB38*	3	141418193	Unrelated	Unrelated	Unrelated	Unrelated	Unrelated	Unrelated	Unrelated	Unrelated	Unrelated	Unrelated	Unrelated	Unrelated	0.959	0.428	0.971	0.423	0.971	0.425	ND	ND

Chr., chromosome; ND, not determined.

You created this PDF from an application that is not licensed to print to novaPDF printer (http://www.novapdf.com)

Table S4 | Linkage disequilibrium coefficients (D' and r^2) of the single nucleotide polymorphisms located in chromosome 4 and 8

	No.	rs ID	Gene	Chr.	Position	Chr. 4																				Chr. 8			
						rs7678436		rs16895802		rs6842303		rs13131350		rs16896276		rs2011603		rs17720281		rs6845999		rs4240326		rs6823268		rs4733724		rs6470764	
						D'	R^2	D'	R^2	D'	R^2	D'	R^2	D'	R^2	D'	R^2	D'	R^2	D'	R^2	D'	R^2	D'	R^2	D'	R^2	D'	R^2
	11	rs7678436	*LCORL*	4	17796343	ND	ND	0.993	0.294	0.989	0.457	0.964	0.702	0.938	0.404	0.932	0.412	Unrelated	Unrelated	Unrelated	Unrelated	Unrelated	Unrelated	Unrelated	Unrelated	Unrelated	Unrelated	Unrelated	Unrelated
	12	rs16895802	*NCAPG*	4	17814266	0.993	0.294	ND	ND	1.000	0.139	0.944	0.352	0.938	0.120	0.939	0.125	Unrelated	Unrelated	Unrelated	Unrelated	Unrelated	Unrelated	Unrelated	Unrelated	Unrelated	Unrelated	Unrelated	Unrelated
	13	rs6842303	*LCORL*	4	17852432	0.989	0.457	1.000	0.139	ND	ND	0.980	0.339	0.917	0.825	0.928	0.849	Unrelated	Unrelated	Unrelated	Unrelated	Unrelated	Unrelated	Unrelated	Unrelated	Unrelated	Unrelated	Unrelated	Unrelated
	14	rs13131350	*LCORL*	4	17875864	0.964	0.702	0.944	0.352	0.980	0.339	ND	ND	0.995	0.343	0.985	0.348	Unrelated	Unrelated	Unrelated	Unrelated	Unrelated	Unrelated	Unrelated	Unrelated	Unrelated	Unrelated	Unrelated	Unrelated
	15	rs16896276	*LCORL*	4	18013533	0.938	0.404	0.938	0.120	0.917	0.825	0.995	0.343	ND	ND	0.991	0.950	Unrelated	Unrelated	Unrelated	Unrelated	Unrelated	Unrelated	Unrelated	Unrelated	Unrelated	Unrelated	Unrelated	Unrelated
FSS	16	rs2011603	*LCORL*	4	18023861	0.932	0.412	0.939	0.125	0.928	0.849	0.985	0.348	0.991	0.950	ND	ND	Unrelated	Unrelated	Unrelated	Unrelated	Unrelated	Unrelated	Unrelated	Unrelated	Unrelated	Unrelated	Unrelated	Unrelated
	17	rs17720281	*HHIP*	4	144622624	Unrelated	Unrelated	Unrelated	Unrelated	Unrelated	Unrelated	Unrelated	Unrelated	Unrelated	Unrelated	Unrelated	Unrelated	ND	ND	0.997	0.983	0.515	0.202	0.247	0.037	Unrelated	Unrelated	Unrelated	Unrelated
	18	rs6845999	*HHIP*	4	144644674	Unrelated	Unrelated	Unrelated	Unrelated	Unrelated	Unrelated	Unrelated	Unrelated	Unrelated	Unrelated	Unrelated	Unrelated	0.997	0.983	ND	ND	0.527	0.209	0.251	0.038	Unrelated	Unrelated	Unrelated	Unrelated
	19	rs4240326	*ANAPC10*	4	144918112	Unrelated	Unrelated	Unrelated	Unrelated	Unrelated	Unrelated	Unrelated	Unrelated	Unrelated	Unrelated	Unrelated	Unrelated	0.515	0.202	0.527	0.209	ND	ND	1.000	0.456	Unrelated	Unrelated	Unrelated	Unrelated
	20	rs6823268	*ANAPC10*	4	145061411	Unrelated	Unrelated	Unrelated	Unrelated	Unrelated	Unrelated	Unrelated	Unrelated	Unrelated	Unrelated	Unrelated	Unrelated	0.247	0.037	0.251	0.038	1.000	0.456	ND	ND	Unrelated	Unrelated	Unrelated	Unrelated
	21	rs4733724	*GSDMC*	8	129711482	Unrelated	Unrelated	Unrelated	Unrelated	Unrelated	Unrelated	Unrelated	Unrelated	Unrelated	Unrelated	Unrelated	Unrelated	Unrelated	Unrelated	Unrelated	Unrelated	Unrelated	Unrelated	Unrelated	Unrelated	ND	ND	1.000	1.000

You created this PDF from an application that is not licensed to print to novaPDF printer (http://www.novapdf.com)

	22	rs6470764	*GSDMC*	8	129713419	Unrelated	Unrelated	Unrelated	Unrelated	Unrelated	Unrelated	Unrelated	Unrelated	Unrelated	Unrelated	Unrelated	Unrelated	Unrelated	Unrelated	Unrelated	Unrelated	Unrelated	Unrelated	Unrelated	Unrelated	1.000	1.000	ND	ND
Control	11	rs7678436	*LCORL*	4	17796343	ND	ND	0.993	0.280	0.971	0.405	0.974	0.649	0.915	0.359	0.916	0.368	Unrelated	Unrelated	Unrelated	Unrelated	Unrelated	Unrelated	Unrelated	Unrelated	Unrelated	Unrelated	Unrelated	Unrelated
	12	rs16895802	*NCAPG*	4	17814266	0.993	0.28	ND	ND	1.000	0.122	0.955	0.379	0.945	0.109	0.947	0.112	Unrelated	Unrelated	Unrelated	Unrelated	Unrelated	Unrelated	Unrelated	Unrelated	Unrelated	Unrelated	Unrelated	Unrelated
	13	rs6842303	*LCORL*	4	17852432	0.971	0.405	1.000	0.122	ND	ND	1.000	0.294	0.920	0.847	0.936	0.859	Unrelated	Unrelated	Unrelated	Unrelated	Unrelated	Unrelated	Unrelated	Unrelated	Unrelated	Unrelated	Unrelated	Unrelated
	14	rs13131350	*LCORL*	4	17875864	0.974	0.649	0.955	0.379	1.000	0.294	ND	ND	1.000	0.294	1.000	0.300	Unrelated	Unrelated	Unrelated	Unrelated	Unrelated	Unrelated	Unrelated	Unrelated	Unrelated	Unrelated	Unrelated	Unrelated
	15	rs16896276	*LCORL*	4	18013533	0.915	0.359	0.945	0.109	0.920	0.847	1.000	0.294	ND	ND	1.000	0.981	Unrelated	Unrelated	Unrelated	Unrelated	Unrelated	Unrelated	Unrelated	Unrelated	Unrelated	Unrelated	Unrelated	Unrelated
	16	rs2011603	*LCORL*	4	18023861	0.916	0.368	0.947	0.112	0.936	0.859	1.000	0.300	1.000	0.981	ND	ND	Unrelated	Unrelated	Unrelated	Unrelated	Unrelated	Unrelated	Unrelated	Unrelated	Unrelated	Unrelated	Unrelated	Unrelated
	17	rs17720281	*HHIP*	4	144622624	Unrelated	Unrelated	Unrelated	Unrelated	Unrelated	Unrelated	Unrelated	Unrelated	Unrelated	Unrelated	Unrelated	Unrelated	ND	ND	0.998	0.986	0.486	0.183	0.208	0.027	Unrelated	Unrelated	Unrelated	Unrelated
	18	rs6845999	*HHIP*	4	144644674	Unrelated	Unrelated	Unrelated	Unrelated	Unrelated	Unrelated	Unrelated	Unrelated	Unrelated	Unrelated	Unrelated	Unrelated	0.998	0.986	ND	ND	0.500	0.191	0.217	0.030	Unrelated	Unrelated	Unrelated	Unrelated
	19	rs4240326	*ANAPC10*	4	144918112	Unrelated	Unrelated	Unrelated	Unrelated	Unrelated	Unrelated	Unrelated	Unrelated	Unrelated	Unrelated	Unrelated	Unrelated	0.486	0.183	0.500	0.191	ND	ND	0.987	0.476	Unrelated	Unrelated	Unrelated	Unrelated
	20	rs6823268	*ANAPC10*	4	145061411	Unrelated	Unrelated	Unrelated	Unrelated	Unrelated	Unrelated	Unrelated	Unrelated	Unrelated	Unrelated	Unrelated	Unrelated	0.208	0.027	0.217	0.030	0.987	0.476	ND	ND	Unrelated	Unrelated	Unrelated	Unrelated
	21	rs4733724	*GSDMC*	8	129711482	Unrelated	Unrelated	Unrelated	Unrelated	Unrelated	Unrelated	Unrelated	Unrelated	Unrelated	Unrelated	Unrelated	Unrelated	Unrelated	Unrelated	Unrelated	Unrelated	Unrelated	Unrelated	Unrelated	Unrelated	ND	ND	1.000	1.000
	22	rs6470764	*GSDMC*	8	129713419	Unrelated	Unrelated	Unrelated	Unrelated	Unrelated	Unrelated	Unrelated	Unrelated	Unrelated	Unrelated	Unrelated	Unrelated	Unrelated	Unrelated	Unrelated	Unrelated	Unrelated	Unrelated	Unrelated	Unrelated	1.000	1.000	ND	ND

Chr., chromosome; ND, not determined.

You created this PDF from an application that is not licensed to print to novaPDF printer (http://www.novapdf.com)

Table S5 | Linkage disequilibrium coefficients (D' and r^2) of the single nucleotide polymorphisms located in chromosome 9, 15 and 18

	No.	rs ID	Gene	Chr.	Position	Chr. 9				Chr. 15										Chr. 18							
						rs10858250		rs12338076		rs2401171		rs10906982		rs7183263		rs11259936		rs4842838		rs4800452		rs4369779		rs4308051		rs8094261	
						D'	R^2	D'	R^2	D'	R^2	D'	R^2	D'	R^2	D'	R^2	D'	R^2	D'	R^2	D'	R^2	D'	R^2	D'	R^2
	23	rs10858250	*QSOX2*	9	136227369	ND	ND	1.000	0.653	Unrelated	Unrelated	Unrelated	Unrelated	Unrelated	Unrelated	Unrelated	Unrelated	Unrelated	Unrelated	Unrelated	Unrelated	Unrelated	Unrelated	Unrelated	Unrelated	Unrelated	Unrelated
	24	rs12338076	*QSOX2*	9	136229894	1.000	0.653	ND	ND	Unrelated	Unrelated	Unrelated	Unrelated	Unrelated	Unrelated	Unrelated	Unrelated	Unrelated	Unrelated	Unrelated	Unrelated	Unrelated	Unrelated	Unrelated	Unrelated	Unrelated	Unrelated
	25	rs2401171	*ADAMTSL3*	15	83888924	Unrelated	Unrelated	Unrelated	Unrelated	ND	ND	0.998	0.993	1.000	1.000	1.000	0.998	1.000	0.998	Unrelated	Unrelated	Unrelated	Unrelated	Unrelated	Unrelated	Unrelated	Unrelated
	26	rs10906982	*ADAMTSL3*	15	83899406	Unrelated	Unrelated	Unrelated	Unrelated	0.998	0.993	ND	ND	0.998	0.993	0.998	0.990	0.998	0.990	Unrelated	Unrelated	Unrelated	Unrelated	Unrelated	Unrelated	Unrelated	Unrelated
	27	rs7183263	*ADAMTSL3*	15	83904289	Unrelated	Unrelated	Unrelated	Unrelated	1.000	1.000	0.998	0.993	ND	ND	1.000	0.998	1.000	0.998	Unrelated	Unrelated	Unrelated	Unrelated	Unrelated	Unrelated	Unrelated	Unrelated
FSS	28	rs11259936	*ADAMTSL3*	15	83911830	Unrelated	Unrelated	Unrelated	Unrelated	1.000	0.998	0.998	0.990	1.000	0.998	ND	ND	1.000	1.000	Unrelated	Unrelated	Unrelated	Unrelated	Unrelated	Unrelated	Unrelated	Unrelated
	29	rs4842838	*ADAMTSL3*	15	83913372	Unrelated	Unrelated	Unrelated	Unrelated	1.000	0.998	0.998	0.990	1.000	0.998	1.000	1.000	ND	ND	Unrelated	Unrelated	Unrelated	Unrelated	Unrelated	Unrelated	Unrelated	Unrelated
	30	rs258324	*CDK10*	16	89687847	Unrelated	Unrelated	Unrelated	Unrelated	Unrelated	Unrelated	Unrelated	Unrelated	Unrelated	Unrelated	Unrelated	Unrelated	Unrelated	Unrelated	Unrelated	Unrelated	Unrelated	Unrelated	Unrelated	Unrelated	Unrelated	Unrelated
	31	rs4800452	*CABLES1*	18	23147647	Unrelated	Unrelated	Unrelated	Unrelated	Unrelated	Unrelated	Unrelated	Unrelated	Unrelated	Unrelated	Unrelated	Unrelated	Unrelated	Unrelated	ND	ND	0.997	0.852	0.997	0.852	0.963	0.822
	32	rs4369779	*CABLES1*	18	23155444	Unrelated	Unrelated	Unrelated	Unrelated	Unrelated	Unrelated	Unrelated	Unrelated	Unrelated	Unrelated	Unrelated	Unrelated	Unrelated	Unrelated	0.997	0.852	ND	ND	1.000	1.000	0.997	0.962
	33	rs4308051	*CABLES1*	18	23155497	Unrelated	Unrelated	Unrelated	Unrelated	Unrelated	Unrelated	Unrelated	Unrelated	Unrelated	Unrelated	Unrelated	Unrelated	Unrelated	Unrelated	0.997	0.852	1.000	1.000	ND	ND	0.997	0.962

You created this PDF from an application that is not licensed to print to novaPDF printer (http://www.novapdf.com)

	34	rs8094261	*CABLES1*	18	23166764	Unrelated	Unrelated	Unrelated	Unrelated	Unrelated	Unrelated	Unrelated	Unrelated	Unrelated	Unrelated	Unrelated	Unrelated	Unrelated	Unrelated	0.963	0.822	0.997	0.962	0.997	0.962	ND	ND
	23	rs10858250	*QSOX2*	9	136227369	ND	ND	1.000	0.667	Unrelated	Unrelated	Unrelated	Unrelated	Unrelated	Unrelated	Unrelated	Unrelated	Unrelated	Unrelated	Unrelated	Unrelated	Unrelated	Unrelated	Unrelated	Unrelated	Unrelated	Unrelated
	24	rs12338076	*QSOX2*	9	136229894	1.000	0.667	ND	ND	Unrelated	Unrelated	Unrelated	Unrelated	Unrelated	Unrelated	Unrelated	Unrelated	Unrelated	Unrelated	Unrelated	Unrelated	Unrelated	Unrelated	Unrelated	Unrelated	Unrelated	Unrelated
	25	rs2401171	*ADAMTSL3*	15	83888924	Unrelated	Unrelated	Unrelated	Unrelated	ND	ND	0.998	0.985	1.000	0.998	1.000	0.998	1.000	0.995	Unrelated	Unrelated	Unrelated	Unrelated	Unrelated	Unrelated	Unrelated	Unrelated
	26	rs10906982	*ADAMTSL3*	15	83899406	Unrelated	Unrelated	Unrelated	Unrelated	0.998	0.985	ND	ND	1.000	0.988	1.000	0.988	1.000	0.985	Unrelated	Unrelated	Unrelated	Unrelated	Unrelated	Unrelated	Unrelated	Unrelated
	27	rs7183263	*ADAMTSL3*	15	83904289	Unrelated	Unrelated	Unrelated	Unrelated	1.000	0.998	1.000	0.988	ND	ND	1.000	1.000	1.000	0.998	Unrelated	Unrelated	Unrelated	Unrelated	Unrelated	Unrelated	Unrelated	Unrelated
**Control**	28	rs11259936	*ADAMTSL3*	15	83911830	Unrelated	Unrelated	Unrelated	Unrelated	1.000	0.998	1.000	0.988	1.000	1.000	ND	ND	1.000	0.998	Unrelated	Unrelated	Unrelated	Unrelated	Unrelated	Unrelated	Unrelated	Unrelated
	29	rs4842838	*ADAMTSL3*	15	83913372	Unrelated	Unrelated	Unrelated	Unrelated	1.000	0.995	1.000	0.985	1.000	0.998	1.000	0.998	ND	ND	Unrelated	Unrelated	Unrelated	Unrelated	Unrelated	Unrelated	Unrelated	Unrelated
	30	rs258324	*CDK10*	16	89687847	Unrelated	Unrelated	Unrelated	Unrelated	Unrelated	Unrelated	Unrelated	Unrelated	Unrelated	Unrelated	Unrelated	Unrelated	Unrelated	Unrelated	Unrelated	Unrelated	Unrelated	Unrelated	Unrelated	Unrelated	Unrelated	Unrelated
	31	rs4800452	*CABLES1*	18	23147647	Unrelated	Unrelated	Unrelated	Unrelated	Unrelated	Unrelated	Unrelated	Unrelated	Unrelated	Unrelated	Unrelated	Unrelated	Unrelated	Unrelated	ND	ND	0.992	0.816	0.996	0.819	0.959	0.787
	32	rs4369779	*CABLES1*	18	23155444	Unrelated	Unrelated	Unrelated	Unrelated	Unrelated	Unrelated	Unrelated	Unrelated	Unrelated	Unrelated	Unrelated	Unrelated	Unrelated	Unrelated	0.992	0.816	ND	ND	1.000	0.996	0.996	0.962
	33	rs4308051	*CABLES1*	18	23155497	Unrelated	Unrelated	Unrelated	Unrelated	Unrelated	Unrelated	Unrelated	Unrelated	Unrelated	Unrelated	Unrelated	Unrelated	Unrelated	Unrelated	0.996	0.819	1.000	0.996	ND	ND	1.000	0.965
	34	rs8094261	*CABLES1*	18	23166764	Unrelated	Unrelated	Unrelated	Unrelated	Unrelated	Unrelated	Unrelated	Unrelated	Unrelated	Unrelated	Unrelated	Unrelated	Unrelated	Unrelated	0.959	0.787	0.996	0.962	1.000	0.965	ND	ND

Chr., chromosome; ND, not determined.

You created this PDF from an application that is not licensed to print to novaPDF printer (http://www.novapdf.com)

Table S6 | Risk genotypes selected for OR calculations

rs ID	Gene	Chr.	Position	Ref	Var	Risk allele homozygote (the risk genotype is coded as "2")	Risk allele heterozygote (the risk genotype is coded as "1")	Non-risk allele homozygote (the non-risk genotype is coded as "0")
rs1046934	*TSEN15*	1	184054395	A	C	AA	AC	CC
rs3791679	*EFEMP1*	2	55869757	C	T	CC	CT	TT
rs3771381	*ZNF638*	2	71333535	T	A	AA	AT	TT
rs10935120	*CEP63*	3	134514250	G	A	AA	AG	GG
rs7632381	*ZBTB38*	3	141387221	T	C	TT	TC	CC
rs13131350	*LCORL*	4	17875864	A	G	GG	GA	AA
rs6845999	*HHIP*	4	144644674	C	T	CC	CT	TT
rs4240326	*ANAPC10*	4	144918112	G	A	GG	GA	AA
rs6470764	*GSDMC*	8	129713419	T	C	TT	TC	CC
rs12338076	*QSOX2*	9	136229894	A	C	AA	AC	CC
rs4842838	*ADAMTSL3*	15	83913372	T	G	GG	GT	TT
rs258324	*CDK10*	16	89687847	C	A	CC	CA	AA
rs4308051	*CABLES1*	18	23155497	G	T	TT	TG	GG

Abbreviations: Chr., Chromosome; Ref, Reference allele; Var, Variant allele.

You created this PDF from an application that is not licensed to print to novaPDF printer (http://www.novapdf.com)

Table S7. Cohorts used for human height GWAS studies

	Reference	Year	PMID number	Population	Cohorts
1	Weedon MN et al., 2008	2008	18391952	European	**1.** The type 2 diabetes (WTCCC-T2D); **2.** Hypertension (WTCCC-HT); **3.** coronary artery (WTCCC-CAD) disease branches; **4.** The national blood service (WTCCC-UKBS); **5.** The Diabetes Genetics Initiative (DGI); **6.** The EPIC Obesity; **7.** The UKT2D GCC, EFSOCH (Exeter Family Study of Childhood Health); **8.** The MRC British Genetics of Hypertension (BRIGHT); **9.** The CoLaus.
2	Gudbjartsson DF et al., 2008	2008	18391951	European	**1.** Icelanders with obesity and cancer; **2.** Dutch individuals with bladder cancer; **3.** Subjects with a premature coronary disease event before 60 years of age; **4.** The Danish Inter99 cohort is a population-based sample of 30- to 60-year-old individuals living in the greater Copenhagen area.
3	Cho YS et al., 2009	2009	19396169	Korean	**1.** The rural Ansung and urban Ansan cohorts with aged 40 to 69; **2.** The Health2 cohort from the Wonju, Pyeong Chang, Gangneung, Geumsan, and Naju regional cohorts in Korea.
4	Soranzo N et al., 2009	2009	19343178	European	**1.** The TwinsUK cohort with adult twin British registry; **2.** The Rotterdam Study (RS) with elderly individuals (age 55 years and over); **3.** The British 1958 Birth Cohort with individuals born within a single week in 1958, and followed periodically from birth to age 44–45 years; **4.** The European Prospective Investigation into Cancer and Nutrition study (EPIC-Norfolk) with aged between 40 and 79 years, resident in Norfolk, UK; **5.** The Chingford Study with residents in North London; **6.** Chuvasha with Caucasian Finno-Ugric speaking population residing in the Chuvasha and Bashkortostan autonomous regions of the Russian Federation; **7.** The Cambridge BioResource (CBR) with pseudo-anonymised DNA samples.

You created this PDF from an application that is not licensed to print to novaPDF printer (http://www.novapdf.com)

5	Lango Allen H et al., 2010	2010	20881960	European	**1.** The primary meta-analysis (Stage 1) included 46 GWA studies of 133,653 individuals; **2.** The in silico follow up (Stage 2) included 15 studies of 50,074 individuals.
6	Kim JJ et al., 2010	2010	19893584	Korean	**1.** The Ansung and Ansan cohorts in the Gyeonggi Province of South Korea.
7	Okada Y et al., 2010	2010	20189936	Japanese	**1.** Japanese subjects from 23 disease sample groups and a healthy control group.
8	Yang J et al., 2012	2012	22426310	European	**1.** The GIANT Consortium
9	Wood AR et al., 2014	2014	25282103	European	**1.** 79 GWAS.
10	He M et al., 2015	2015	25429064	European, Asian	**1.** the 11 GWAS with East Asian ancestry in Stage 1; **2.** East Asian ancestry from five additional GWAS.
11	Chan Y et al., 2015	2015	25865494	Various	**1.** Atherosclerosis Risk in Communities; **2.** Cardiovascular Health Study; **3.** Coronary Artery Risk Development in Young Adults; **4.** Framingham Heart Study; **5.** 1958 British Birth cohort; **6.** Avon Longitudinal Study of Parents and Children.

You created this PDF from an application that is not licensed to print to novaPDF printer (http://www.novapdf.com)

Figure S1

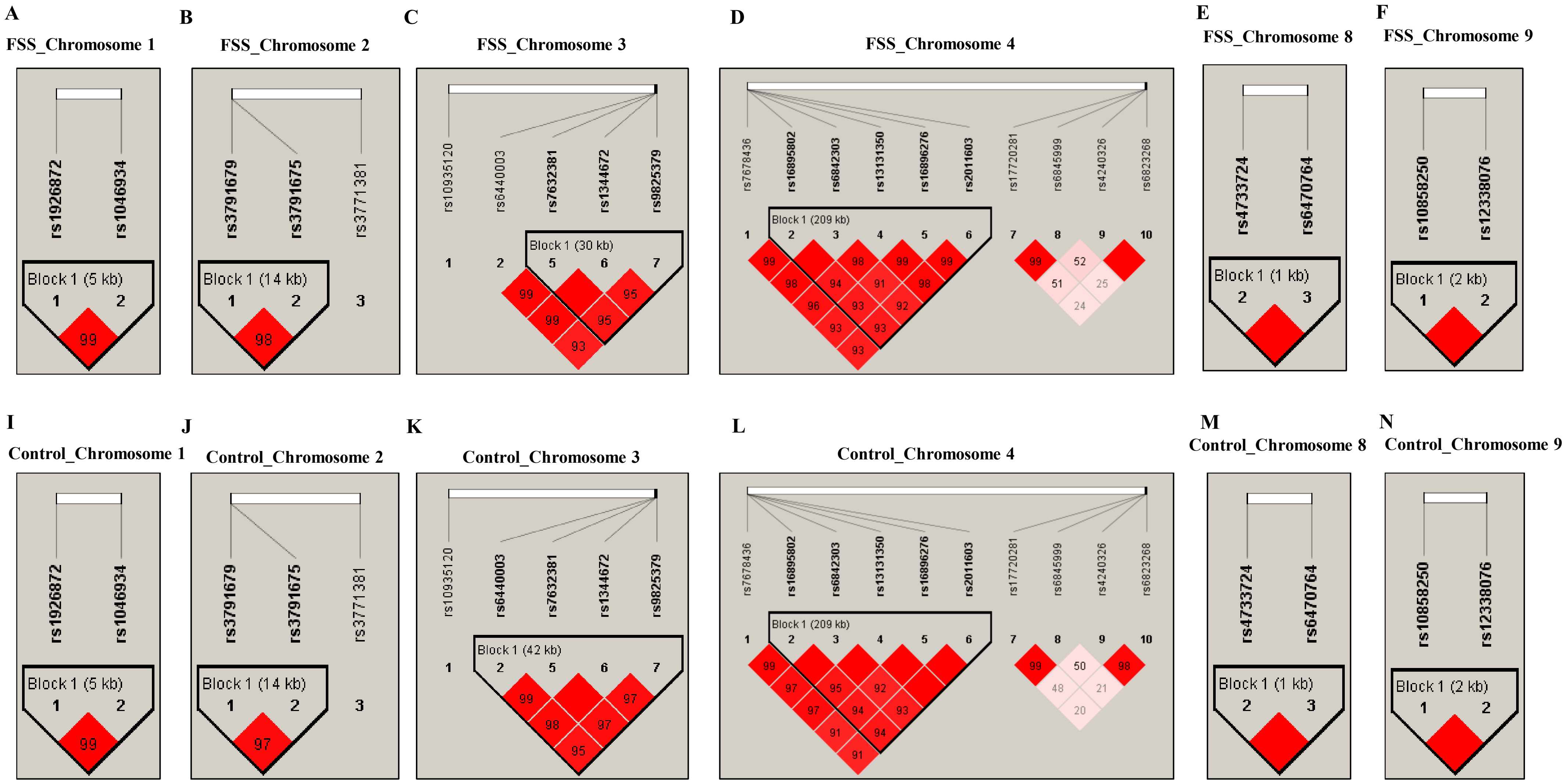

You created this PDF from an application that is not licensed to print to novaPDF printer (http://www.novapdf.com)

Figure S1

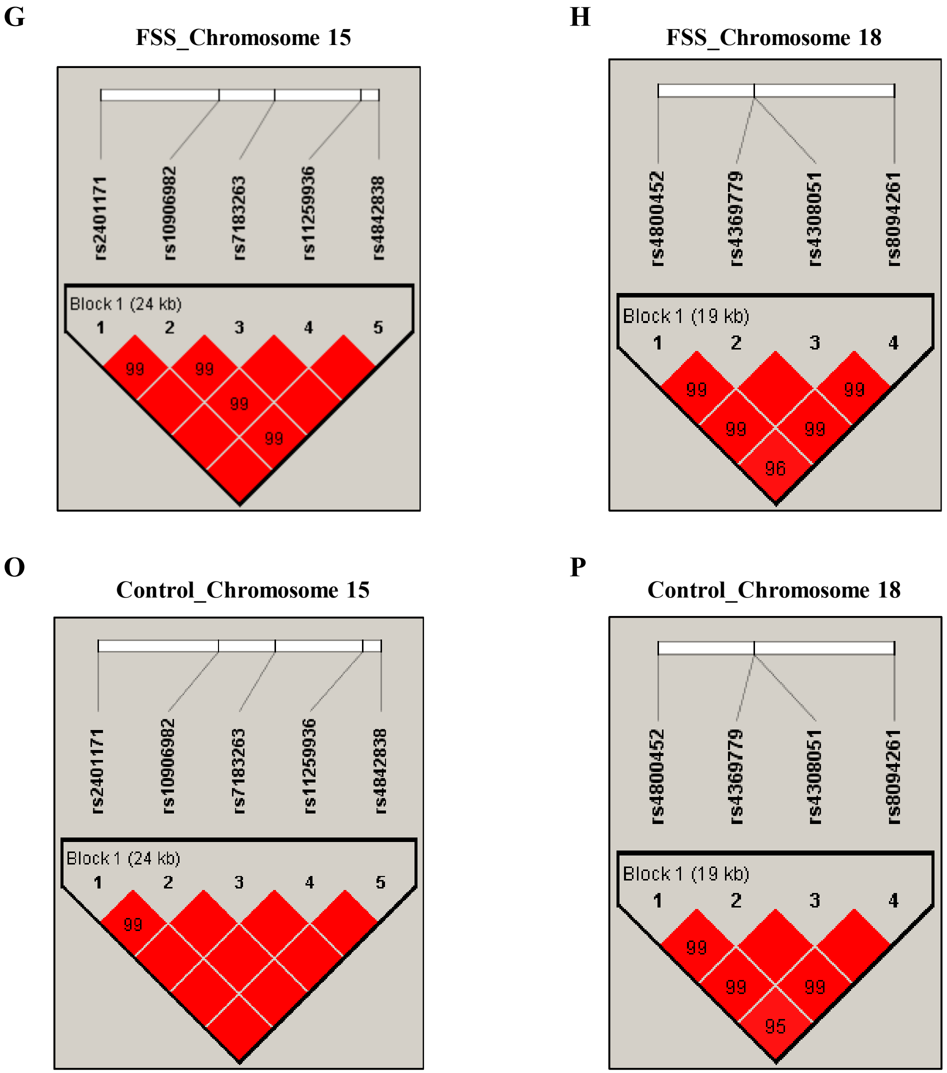

Figure S1. Results of a single-nucleotide polymorphism (SNP) association study of the genetic SNPs identified from GWAS studies of human height in FSS and healthy individuals from the general population of Taiwan who were of Han Chinese ethnic background. Haplotype blocks with different chromosome positions for the 978 FSS cases (A-H) and 1,129 control subjects (I-P) constructed according to the confidence interval approach using Haploview software[50, 51, 52]. Red indicates linkage disequilibrium (D' = 1, logarithm of odds [LOD] ≥2); pink indicate evidence of recombination (D' < 1, LOD <2).

You created this PDF from an application that is not licensed to print to novaPDF printer (http://www.novapdf.com)

Figure S2

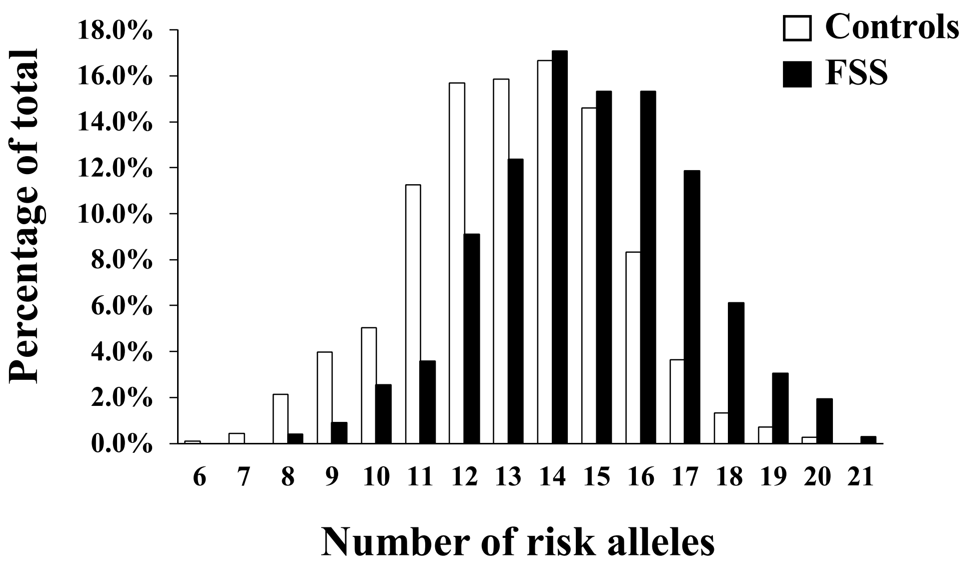

Figure S2. Distribution of the number of risk alleles.

You created this PDF from an application that is not licensed to print to novaPDF printer (http://www.novapdf.com)

Figure S3

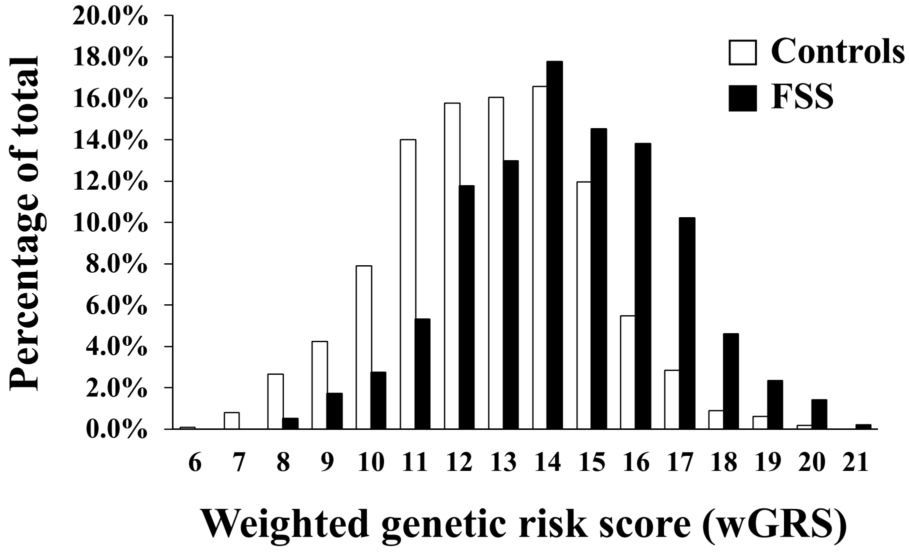

Figure S3. Distribution of the number of weighted genetic risk score.

You created this PDF from an application that is not licensed to print to novaPDF printer (http://www.novapdf.com)

Figure S4

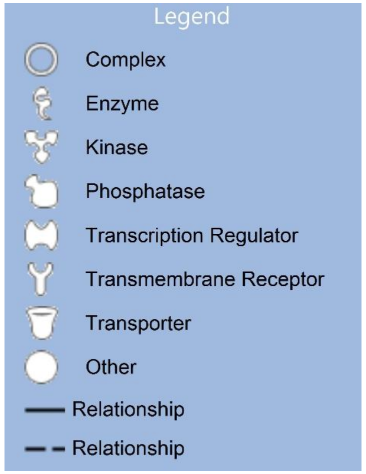

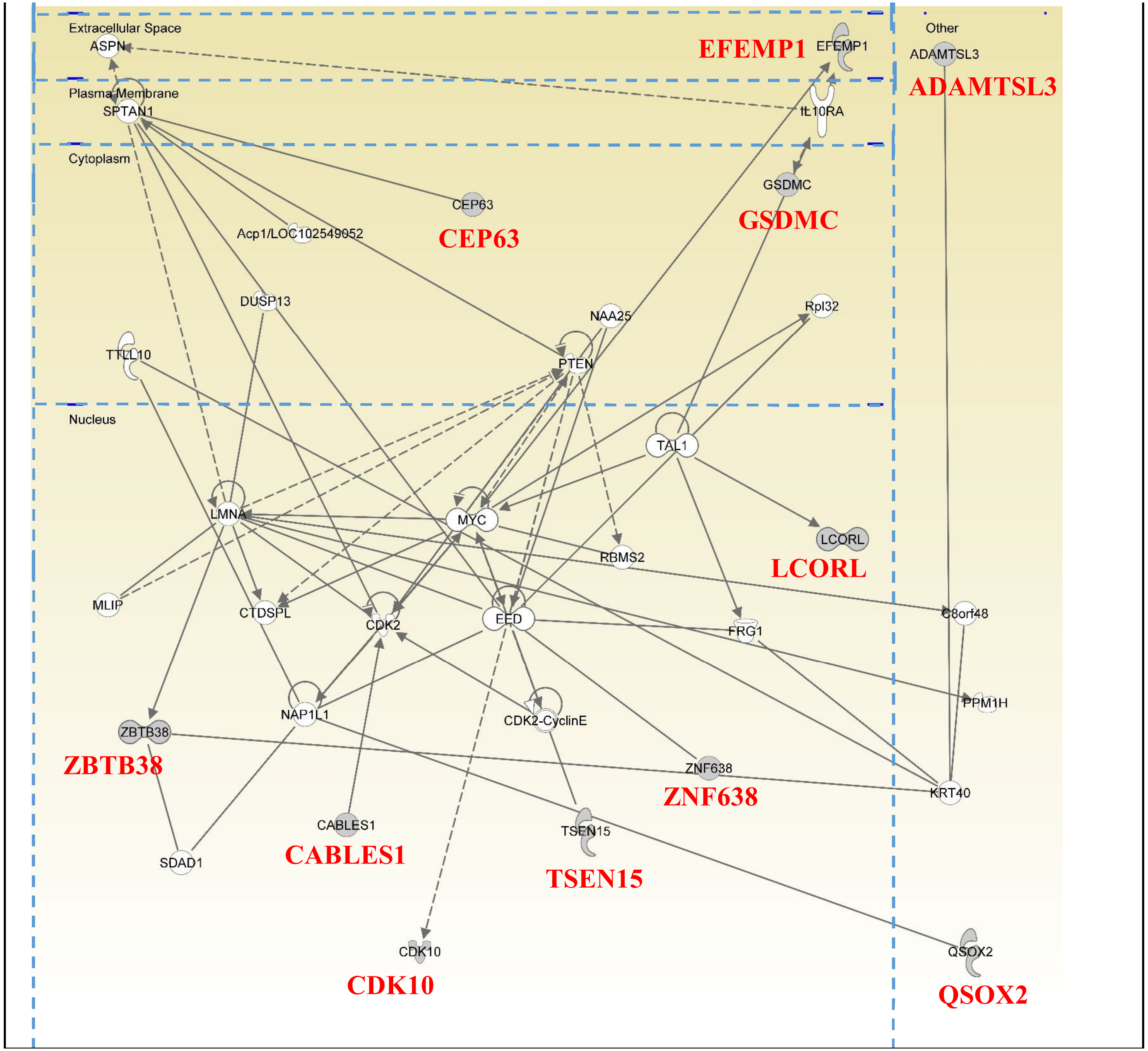

Figure S4. Putative gene network derived from Ingenuity Pathway Analysis (IPA) software. IPA network analysis identified a single cluster of 35 genes that includes 11 associated genes discovered in this study. The lines between genes represent known interactions (solid lines represent direct interactions; dashed lines represent indirect interactions). Each gene is displayed using various shapes that represent the functional class of the gene product, as indicated in the legend.

You created this PDF from an application that is not licensed to print to novaPDF printer (http://www.novapdf.com)
